# A Scalable System for Production of Functional Pancreatic Progenitors from Human Embryonic Stem Cells

**DOI:** 10.1371/journal.pone.0037004

**Published:** 2012-05-18

**Authors:** Thomas C. Schulz, Holly Y. Young, Alan D. Agulnick, M. Josephine Babin, Emmanuel E. Baetge, Anne G. Bang, Anindita Bhoumik, Igor Cepa, Rosemary M. Cesario, Carl Haakmeester, Kuniko Kadoya, Jonathan R. Kelly, Justin Kerr, Laura A. Martinson, Amanda B. McLean, Mark A. Moorman, Janice K. Payne, Mike Richardson, Kelly G. Ross, Eric S. Sherrer, Xuehong Song, Alistair Z. Wilson, Eugene P. Brandon, Chad E. Green, Evert J. Kroon, Olivia G. Kelly, Kevin A. D’Amour, Allan J. Robins

**Affiliations:** 1 Viacyte, Inc., Athens, Georgia, United States of America; 2 Viacyte, Inc., San Diego, California, United States of America; 3 Department of Biochemistry and Molecular Biology, University of Georgia, Athens, Georgia, United States of America; University of British Columbia, Canada

## Abstract

Development of a human embryonic stem cell (hESC)-based therapy for type 1 diabetes will require the translation of proof-of-principle concepts into a scalable, controlled, and regulated cell manufacturing process. We have previously demonstrated that hESC can be directed to differentiate into pancreatic progenitors that mature into functional glucose-responsive, insulin-secreting cells *in vivo*. In this study we describe hESC expansion and banking methods and a suspension-based differentiation system, which together underpin an integrated scalable manufacturing process for producing pancreatic progenitors. This system has been optimized for the CyT49 cell line. Accordingly, qualified large-scale single-cell master and working cGMP cell banks of CyT49 have been generated to provide a virtually unlimited starting resource for manufacturing. Upon thaw from these banks, we expanded CyT49 for two weeks in an adherent culture format that achieves 50–100 fold expansion per week. Undifferentiated CyT49 were then aggregated into clusters in dynamic rotational suspension culture, followed by differentiation en masse for two weeks with a four-stage protocol. Numerous scaled differentiation runs generated reproducible and defined population compositions highly enriched for pancreatic cell lineages, as shown by examining mRNA expression at each stage of differentiation and flow cytometry of the final population. Islet-like tissue containing glucose-responsive, insulin-secreting cells was generated upon implantation into mice. By four- to five-months post-engraftment, mature neo-pancreatic tissue was sufficient to protect against streptozotocin (STZ)-induced hyperglycemia. In summary, we have developed a tractable manufacturing process for the generation of functional pancreatic progenitors from hESC on a scale amenable to clinical entry.

## Introduction

Since the development of the Edmonton protocol [1], clinical islet transplantation has proven the feasibility of a curative treatment for type 1 diabetes, a debilitating disease caused by autoimmune destruction of pancreatic β-cells. Replacing lost β-cell function is an effective therapeutic strategy, but the shortage of pancreata available for islet isolation places serious constraints on this intervention option [2]. Therefore, efforts are focused on developing a larger scale source of pancreatic cells with clinical utility. Human embryonic stem cells (hESC) are an attractive option due to their vast proliferative and differentiation potential [3], [4]. Using a step-wise protocol, we have demonstrated that hESC can be directed to differentiate to a mixed population comprised of pancreatic endoderm (PE) and poly-hormonal endocrine cells [5], [6], [7]. Implanted PE gives rise to functioning islet-like structures *in vivo* through a mechanism that appears to primarily involve the *de novo* commitment of pancreatic progenitors to the endocrine lineages followed by further maturation to glucose-responsive β-cells. Such grafts are therefore capable of sensing blood glucose, responding with metered release of processed human insulin, and protecting against streptozotocin (STZ)-induced hyperglycemia in mice [6], [7]. Implantation of enriched populations has demonstrated that PE, defined as chromogranin A (CHGA) negative and NKX6-1/PDX1 co-positive, and not the poly-hormonal endocrine cells, are progenitors of these islet-like structures [7].

The production of hESC-derived pancreatic progenitors offers a promising approach to circumvent issues with the supply of clinical cadaveric islets [3]. Nonetheless, bringing a cell therapy to the clinic requires developing manufacturing processes that consistently generate pancreatic populations that are functional and safe, eventually at a scale sufficient to produce many human doses in single manufactured lots. Thus far, protocols for generating hESC-derived pancreatic cells with proven utility to regulate blood glucose *in vivo* have only been described on a small scale using adherent cell culture formats that exhibit variable cell compositions [6], [7]. While other candidate pancreatic lineages have been derived from hESC [8], [9], [10], [11], none have demonstrated robust post-engraftment function *in vivo*, as defined by both long-term glucose-responsive human C-peptide secretion and protection against STZ-induced hyperglycemia [12], [13], [14]. Without demonstrated function in animal models, it is difficult to gauge the scalability, or clinical potential, of these alternate protocols.

Limitations in the methods for cryopreservation, expansion, and directed differentiation all restrict the ability to generate large amounts of functional pancreatic progenitors from hESC. Furthermore, it is not yet feasible to expand a stable endodermal progenitor that maintains a comparable differentiation potential *in vivo*. As we typically observe a near 1∶1 ratio of starting hESC to differentiated end-stage cells [5], our strategy to increase the amount of implantable material produced has therefore concentrated on efficient expansion of hESC, followed by their differentiation to pancreatic progenitors en masse.

With a doubling time of approximately 24 hrs [15], hESC exhibit a remarkable capacity for expansion in culture if harnessed effectively. As a proof-of-concept for this capacity, we have previously demonstrated that feeder-free conditions using defined media can support single cell passaging and bulk culture of hESC [16]. A single batch of >1×10^10^ BG02 hESC was produced that represented an expansion of four orders of magnitude in 6 passages [16]. Critical for the progression of hESC-based technology to clinical trials is a demonstration of comparable scalability using cGMP-compliant manufacturing processes with appropriately developed reagents. Improvements that enhance expansion efficiencies will also save time and produce cost savings, as well as minimize the potential for population drift over time spent in culture [17]. Importantly, robust scaling and cryopreservation of hESC will enable the established strategy of master- and working-cell banks (MCB and WCB, respectively) to be employed, and will provide defined and consistent material for product manufacture.

In this report, we describe coupled processes that permit scaled production of hESC and thereafter, pancreatic progenitors. A feeder-free culture system was developed for expansion of the CyT49 hESC line (NIH registration number: 0041. [6]) and the generation of large-scale, single cell MCB and WCB of CyT49 under cGMP. We also developed a rotational suspension-based differentiation method. The reproducibility of this protocol was demonstrated by performing repeated differentiation runs with consistent pancreatic cell compositions and production of glucose-responsive insulin-secreting cells after maturation of progenitors *in vivo* in many cohorts of mice. Levels of human insulin release were sufficient to protect against STZ-induced hyperglycemia, and implants were capable of prolonged engraftment. Moreover, as a proof of scalability, we report the production of 3.3×10^9^ pancreatic cells in a single manufactured lot with only four passages from a single thawed vial of a high-density CyT49 bank. Our suspension-based approach coupled with scaled hESC culture, represents an integrated and robust methodology that will be the foundation for manufacturing pancreatic progenitors for clinical trials.

## Results

### Scalable Conditions for Adherent hESC and cGMP Cell Banking

We have developed a feeder-free culture method for hESC, to enable scaled expansion of adherent cultures of the CyT49 cell line, and the cGMP production of cell banks from single-cell suspensions. The media was composed of DMEM/F12 supplemented with Xeno-free KnockOut™ Serum Replacement, recombinant heregulin-1β and activin A, a formulation providing self-renewal signaling similar to the defined media we described previously [16], [18]. Cell attachment was facilitated using a soluble activity present in human serum, which was added to the media on the first day of each passage. Undifferentiated CyT49 cells could be maintained under these conditions, using serial passaging with Accutase™ (Fig. S1), which was the approach taken for all the experiments described here. Accutase was the only reagent in these conditions with xeno-sourced components. Optimization of our conditions increased expansion efficiencies by defining plating densities for either a three- or four-day interval of culture, and refinements to feeding volumes and schedules. We found that tight control over cell dissociation, increasing the volume of media used in each subsequent day of culture and feeding with fresh media for several hrs on the day of passage were instrumental in maintaining cell viabilities and plating efficiencies of >90% (Fig. S2). The method was applied to the routine culture of CyT49 in large T-flasks, and cell factories, achieving 50–100 fold expansion per week (Table S1).

A series of CyT49 cell banks were generated using this scalable system (Table 1, Fig. S3A). Cryopreservation of hESC has traditionally been a problem, characterized by poor viability and/or plating efficiency of thawed cell clusters [19]. The original cGMP hESC banks of CyT49, MCB1 and WCB1, were made with cell clusters, which are not amenable to rapid, or scalable, post-thaw expansion and differentiation. Banks of dissociated CyT49 cells were therefore prepared to demonstrate that cultures could be successfully expanded on a large scale and cryopreserved as single-cell suspensions, and that high thaw viabilities and plating efficiencies could be achieved in such a format. The research cell banks RCB-D and RCB-Dw were prepared from cultures of CyT49 that had been previously adapted to culture in defined media [18]. Thaw viability of RCB-D was 93.1±2.6% (mean ± SD, n = 5 vials) and these cultures averaged a 3.6-fold expansion within 4 days, by the first post-thaw passage. RCB-Dw was generated after an additional 3 passages from a thawed vial of RCB-D, and exhibited a thaw viability of 93.9±3.2% (n = 3 vials). Cell counting after 24 hrs of culture, which takes into account plating efficiency, proliferation and cell death, indicated that 93.4±6.4% of the plated cell number remained (n = 3). We observed similar thaw and expansion efficiencies from a single cell bank of the BG02 cell line [16]. Here we confirm and extend these findings to demonstrate the ability to cryopreserve banks of hESC in single-cell format using a classic master and working cell bank strategy. Next, separate thaws from MCB1 were transitioned to single-cell conditions, expanded and cryopreserved as RCB-E and RCB-G, in order to trial and optimize the procedure for high-density banking at 10^7^ cells/vial. Thaw viabilities of 87.8±5.7% and 85.5±2.1% (n = 3 vials) were observed for RCB-E and RCB-G, respectively, and 24 hr cell counts were 98.2±13.8% and 102.3±12.8% of plated cells, respectively. Following these pilot studies, single cell MCB and WCB were generated according to cGMP for eventual product manufacturing (Table 1). Two vials each of MCB1 and WCB1 were thawed, transitioned to feeder-free conditions in xeno-free growth media, expanded and cryopreserved independently as MCB3, MCB4 and MCB5. While karyotypically normal prior to cryopreservation, a fourth expansion (MCB6) was disqualified after mosaic aneuploid populations were observed within four passages of thawing banked cells. After *in vitro* qualification and *in vivo* functional evaluation, WCB4B was produced from MCB4 according to cGMP. Thawed cultures from these banks were shown to exhibit a normal karyotype by G-banding (Table 1). Further examination indicated that banked cells were undifferentiated and retained characteristics of hESC such as an undifferentiated morphology and markers of pluripotent cells (Fig. S3B–D). In order to demonstrate the capacity for rapid bulk-expansion of CyT49 cultures, we produced 2.7×10^9^ cells from a single vial of 10^7^ cryopreserved cells in standard tissue culture T-flasks and cell stacks in a 2-week period (Table S1, Table S2 Expt #21). Only ∼25% of the potential expansion capacity of this culture was utilized, indicating that as many as 1–2×10^10^ CyT49 cells could have been readily generated within the same time frame.

**Table 1:** Colony cluster banks, single cell research-, master-, and working-cell banks of CyT49.

Bank	p#	Hrv	#V	#/vial	T%	Karyotype post thaw	
MCB1^ C G^	p9	30×60 mm plates	53	na	na	M	46,XY [20]
WCB1^ C G^	p14	27×60 mm plates	75	na	na	M	46,XY [20]
RCB-D	p21	1.4×10^8^	90	1.5×10^6^	93.1	M	46,XY [20]
RCB-Dw	p24	6.9×10^8^	69	1.2×10^6^	93.9	1	46,XY [19], 46,XY,del(2)(p21q31)[1*]
						2	46,XY [19], 43,XY,−18, −20, −22[1*]
RCB-E	p20	4.3×10^8^	43	1×10^7^	87.8	1	46,XY [19], 46,XY,t(8;13)(p11.1;q11)[1*]
						2	46,XY [19], 46,XY,+i(1)(q10)[1*]
						3	46,XY [20]
						4	46,XY [20]
						5	46,XY [20]
RCB-G	p19	5×10^8^	50	1×10^7^	85.5	1	46,XY [19], 46,XY,der(1)t(1;14)(q44;q11.2)[1*]
MCB3 ^G ¶^	p23	1.2×10^9^	118	1×10^7^	89.3	1	46,XY [20]
						2	46,XY [20]
						3	46,XY [20]
MCB4^ G ¶^	p23	1.6×10^9^	157	1×10^7^	92.3	1	46,XY [20]
						2	46,XY [20]
						3	46,XY [20]
MCB5^ G Ω^	p22	1.1×10^9^	108	1×10^7^	93.4	1	46,XY [29], 46,XY,i(12)(p10)[1*]
						2	46,XY [20]
						3	46,XY [20]
WCB4B^ G^	p27	2.6×10^9^	267	1×10^7^	93	1	46,XY [20]
						2	46,XY [20]
						3	46,XY [20]

p#: passage number from derivation of the line. Hrv: total cells harvested. #V: number of vials frozen. #/vial: cells/vial. T%: thaw viability (%). na: not available. ^C^: bank of colony clusters. ^G^: cGMP manufacture. ^Ω^: derived from MCB1. ^¶^: derived from WCB1. Karyotype analyses indicates the thaw number (left column), and the number of nuclei (bracketed) for each class of result. M: multiple thaws. *: non-clonal, deemed technical.

### Reproducible Production of Functional Pancreatic Progenitors with a Scalable Suspension-Based Manufacturing Process

**Figure 1:**
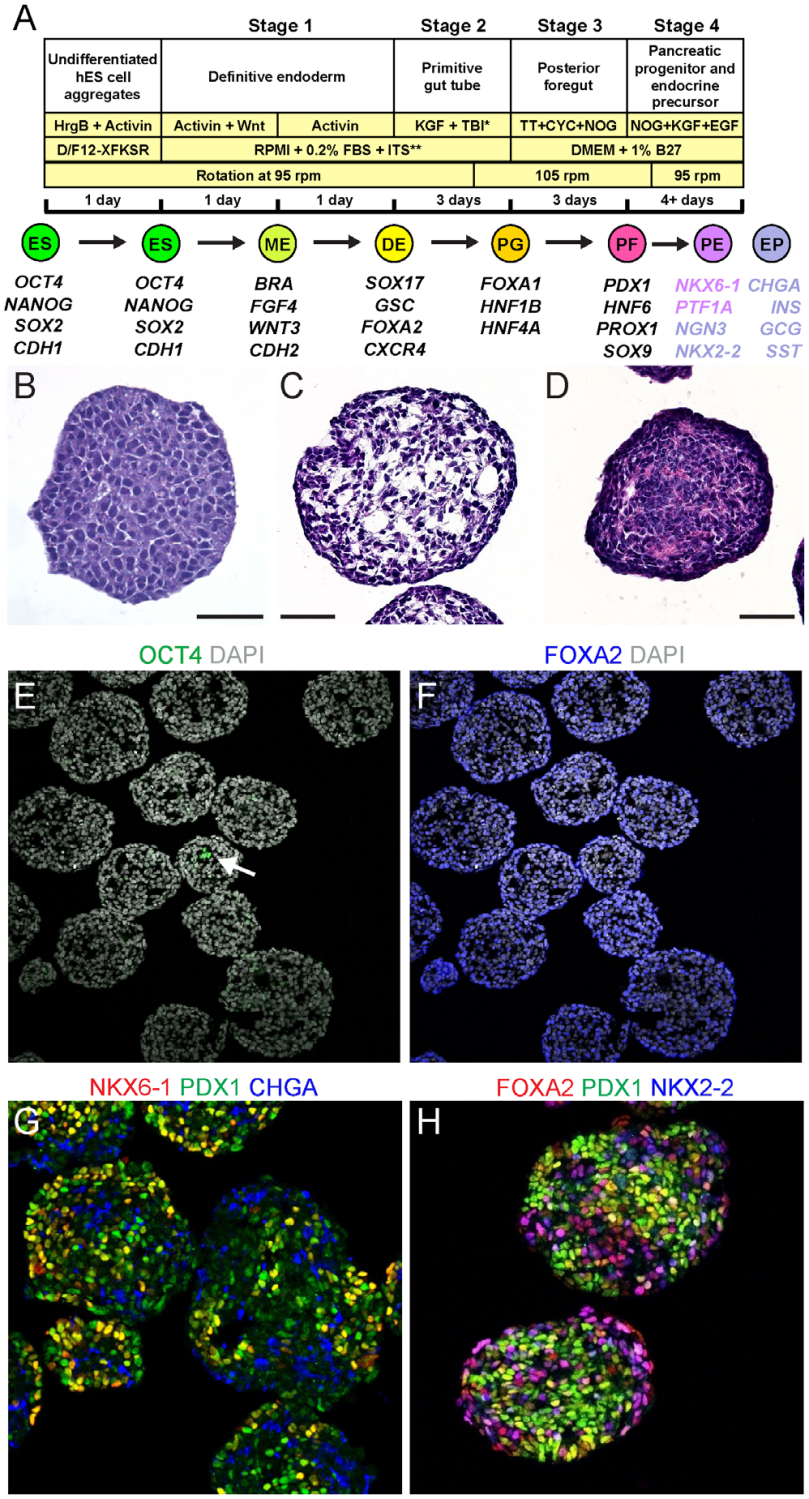
Directed pancreatic differentiation of CyT49 in suspension culture. (A) Schematic representation of aggregation and the four-stage differentiation protocol from hESC (ES) to mesendoderm (ME), definitive endoderm (DE), primitive gut tube (PG), posterior foregut (PF), and a mixed population comprising primarily of pancreatic endoderm (PE) and endocrine precursor/endocrine cells (EP). Culture conditions, timing and rotation speeds are indicated. Markers used to identify the different stages are shown.*TBI: TGF-β RI kinase Inhibitor IV, first day of Stage-2 only. **ITS: Insulin-Transferrin-Selenium used at different concentrations in Stage-1 and -2. HrgB, heregulin 1β; D/F12, DMEM/F12; TT, TTNPB; CYC, cyclopamine; NOG, noggin. (B,C,D) Hematoxylin and eosin staining of sections of CyT49 aggregates after (B) culture in StemPro medium for 2 days (paraffin section), or (C) at d5, or (D) at d12 of differentiation (frozen sections). Scale bars, 50 µm. (E) Immunofluorescence analysis of sections of d5 aggregates stained with OCT4/DAPI, or (F) FOXA2/DAPI. A single cluster of ∼four OCT4^+^ nuclei within the field of view is indicated (arrow). Imaged with a 20× objective. Immunofluorescence analysis of d12 aggregates for (G) NKX6-1, PDX1 and CHGA expression, and (H) FOXA2, PDX1 and NKX2-2 expression. Imaged with a 40× objective. DAPI, 4′,6-diamidino-2-phenylindole.

To circumvent surface-area constraints of adherent-based systems and permit manufacturing-scale culture and differentiation, we developed suspension methodologies for the production of pancreatic progenitors. Adherent hESC were dissociated to single cells and aggregated in suspension to generate three-dimensional spheres of undifferentiated cells (Fig. 1, Fig. S4). Cells were seeded at 1×10^6^ cells/mL in ultra-low adhesion 6-well dishes and incubated under rotation on an orbital platform. Principles of bioengineering design were used to examine factors that influenced adhesion and aggregation in suspension [20], such as seeding density, volume, rotational radius and speed, collision frequency and shear rate, in a systematic optimization of aggregation (not shown). Under optimal conditions, aggregates formed by self-association overnight, generating spherical clusters with diameters of 100–200 µm. Modeling of diffusion rates suggests that aggregates of this size would not be expected to be substantially impacted by mass transfer limitations [21]. Efficient rates of incorporation into aggregates were typically observed, in the range of 75% of input cells after 24 hrs (Fig. S4). Aggregates displayed high uniformity and lacked cavitation, cystic structures, or cellular layering that would indicate spontaneous differentiation. The completely undifferentiated nature of these aggregates was indicated by the maintenance of uniform expression of hESC markers and absence of gene expression for differentiated phenotypes (Figs. S4, S6). Undifferentiated cultures of hESC could be maintained in suspension via serial passaging of aggregates (Fig. S4).

We adapted our previously reported pancreatic differentiation protocols [6], [7], to the suspension system and optimized the methodology to enable efficient and large-scale differentiation of the CyT49 cell line (Fig. 1). Differentiation of CyT49 was initiated the day after hESC aggregation, and was typically carried out over 12 days (Fig. S5), although in some cases Stage-4 was extended up to 8 days (d16). As before, the procedure entailed directing the cells through successive intermediates including mesendoderm (d1), definitive endoderm (d2), nascent gut endoderm (d5), posterior foregut endoderm (d8), and pancreatic endoderm (PE) with endocrine precursors (d12), en route to robust hormone expression (d16) (Fig. 1A). The suspension differentiation protocol involved only a few modifications from our previous publications [5], [6], [7]. The TGF-β RI kinase Inhibitor IV was included during Stage-2, and retinoic acid was replaced with a more stable retinoid analog, TTNPB (3 nM), during Stage-3. The growth factors KGF (50 ng/mL) and EGF (50 ng/mL) were added to Stage-4 to preserve cell mass. Noggin (50 ng/mL) was also included at Stage-4. Using TGFβ inhibitors, other reports have described increased production of endocrine cells, principally poly-hormonal in nature [22], [23], an effect we have also observed (data not shown). Sectioning of undifferentiated aggregates and aggregates at the end of Stages-2 and -4, confirmed that distinct transitions in cellular architecture occurred during differentiation (Fig. 1B–D).

Using this modified protocol, a series of thirty-seven independent differentiation runs were carried out to assess the reproducibility of this scalable manufacturing process. To minimize technical dissimilarity from batch-to-batch and between multiple handlers, tight controls on cell culture variables were implemented. Each process began with a thaw from one of the large-scale single cell CyT49 hESC banks, followed by acute expansion in adherent culture. These scaled cultures maintained a normal karyotype (Table S2). Starting from expanded undifferentiated cultures of typically 6.6×10^8^ CyT49 cells, hESC were aggregated and differentiated en masse.

Extensive analyses of gene expression and cellular composition were used to characterize differentiating aggregates. Rare OCT4-immunoreactive cells persisted until day 5 of differentiation at the latest, and at this time point were only detected in small pockets of cells within aggregates that consisted predominantly of OCT4^−/^FOXA2^+^ target endoderm cells (Fig. 1E, F). These OCT4^+^ cells also expressed SOX2, but not NANOG (not shown), indicating that they were not likely to be pluripotent stem cells. By the end of Stage-4, nuclear-localized NKX6-1 and PDX1 transcription factors were detected in aggregates, marking PE in a mixed pancreatic population that also included NKX2-2^+^ and CHGA^+^ endocrine cells (Fig. 1G, H). Some non-endocrine PDX1^+^ cells that did not express NKX6-1 were present as well; it is not known if these cells represent an earlier stage of PDX1^+^ pancreatic progenitors, or a non-pancreatic lineage such as posterior stomach, or duodenum [6]. Unlike NKX6-1^+^/PDX1^+/−^ PE, which has demonstrated islet cell neogenesis potential *in vivo* [7], the potential for NKX6-1^−/^PDX1^+^ endoderm to contribute to functional β-cell mass *in vivo* is at this point unclear.

**Figure 2:**
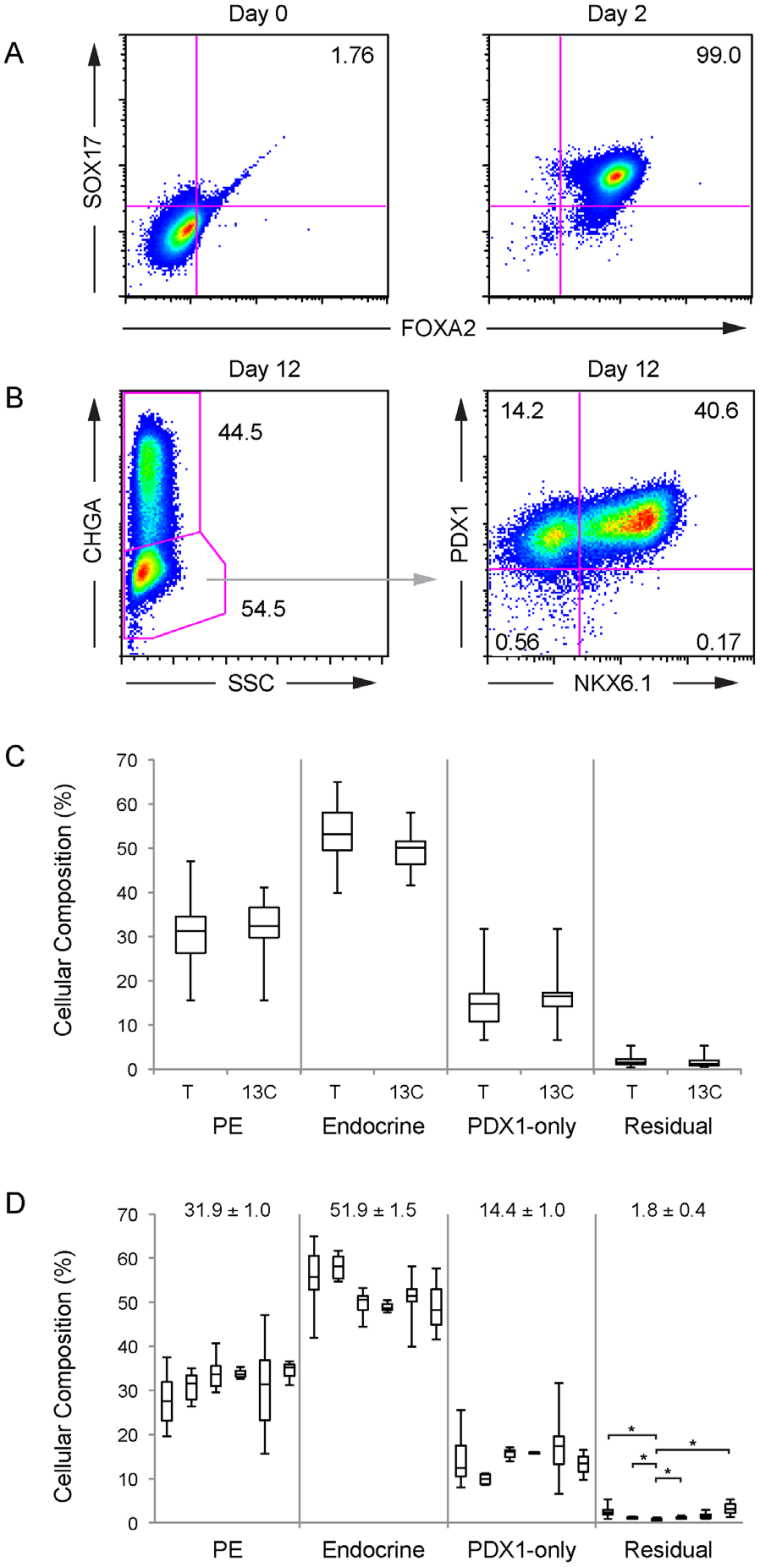
Cellular composition of pancreatic differentiation runs. (A) Flow cytometric analyses of representative d0 undifferentiated (left), and d2 DE populations (right), co-stained with anti-SOX17 and anti-FOXA2. The percentages of total intact double-positive cells are indicated. (B) Flow cytometric analysis of a d12 differentiated population (scaled differentiation run, Expt #21) co-stained with anti-CHGA, anti-NKX6-1 and anti-PDX1. The analyses were performed by first gating on the CHGA^-^ population, then plotting NKX6-1/PDX1 expression. Percentages of total intact cells for each cell subset are indicated. (C) The cellular composition of PE (CHGA^−/^NKX6-1^+^/PDX1^+/−^), endocrine (CHGA^+^/NKX6-1^+/−/^PDX1^+/−^), PDX1-only endoderm (CHGA^−/^NKX6-1^−/^PDX1^+^) and residual (CHGA^−/^NKX6-1^−/^PDX1^−^) populations for all 37 scaled differentiation runs (T: total, n = 49 analyses) and the selected processes (13C: Table S2 Expt #18–21, 25–30, 35–37. n = 17 analyses) are plotted. The 13C group of differentiation runs were all performed within an 11 month period. The box plots show the median, second and third quartile (box), max and min values for each data set. The means within each group were not statistically different. (D) Pancreatic composition of differentiation runs arranged by CyT49 cell bank: (left to right) RCB-D (n = 19), MCB3 (n = 6), MCB4 (n = 7), MCB5 (n = 3), RCB-Dw (n = 11) and WCB4B (n = 3). The mean ± SEM (%) for each population is indicated at the top. Statistically significant differences are indicated (*: p<0.01).

We also assessed the cellular compositions of undifferentiated d0 aggregates, and at day 2, day 12 and day 16 of differentiation using flow cytometry. The proportion of SOX17^+^/FOXA2^+^ cells increased from a rare 1.76% at d0, to 99.0% of the population after two days of differentiation, indicating highly efficient specification of definitive endoderm (Fig. 2A). The composition of each of the thirty-seven scaled differentiation runs was examined at Stage-4 (n = 45 d12, n = 4 d16 analyses), demonstrating that a large majority of differentiated cells expressed CHGA, NKX6-1, or PDX1 (Fig. 2B, Table S2). Differentiation runs contained 40–65% immature endocrine or poly-hormonal cells (CHGA^+^), 16–47% PE cells (CHGA^−/^NKX6-1^+^), and 7–32% PDX1-expressing endoderm (CHGA^−/^NKX6-1^−/^PDX1^+^). Typically, fewer than 2% of cells failed to be demarcated by any of these three markers, compared to the ∼10% that were observed in the best examples of our previous plate-based adherent differentiation methodology [7]. While the proportions of distinct cell subsets varied to some extent among differentiation runs, the low frequency of unidentified cell types (CHGA^−/^NKX6-1^−/^PDX1^−^) was consistently observed. Differentiation runs extended to d16 did not differ markedly in cellular composition from d12 populations (Table S2), although immuofluorescent analyses confirmed that expression of endocrine hormones became more robust (data not shown).

To compare the outcomes of the differentiation process from different banks, as well as to model lot-to-lot variation, we focused a more detailed analysis on a selected group (n = 13 differentiation runs, 3 banks, 17 cytometric analyses). Included in this group was a batch that utilized 2.7×10^9^ hESC and yielded 3.3×10^9^ Stage-4 cells, as a demonstration of the scalability of this process (Table S2 Expt #21). Two groups of processes that were performed semi-consecutively, termed “13C”, were selected to investigate lot-to-lot consistency (Table S2). Group 1 consisted of: MCB4 expt #18–20, RCB-Dw expt #25–30 (9 sequential runs); and Group 2 of: MCB4 expt #21, WCB4B expt #35–37 (4 sequential runs). Box-plots of the cellular composition of all the manufacturing runs (Table S2) compared to the 13C group, demonstrated a robust and consistent distribution of pancreatic lineages in sequential experiments (Fig. 2C). A bank comparison confirmed that such consistency was achieved from 6 different CyT49 banks (Fig. 2D), indicating excellent process control over pancreatic specification. An average of 51.9±1.5 (mean ± SEM)% immature endocrine or poly-hormonal cells, 31.9±1.0% PE cells, and 14.4±1.0% PDX1-expressing endoderm were observed, with only 1.8±0.4% of cells not positively stained for any of these markers (Fig. 2D). The means within the PE, endocrine, or PDX-1 only groups were not statistically different, whereas the mean of the residual population (CHGA^−/^NKX6-1^−/^PDX1^−^) in the differentiation runs from MCB4 was lower than those from the RCB-D, MCB3, RCB-Dw and WCB4B banks (p<0.01). While highlighting change in only a minor fraction of the population, it suggests that the four MCB4-derived differentiation runs exhibited the tightest technical control over target lineage specification.

**Figure 3:**
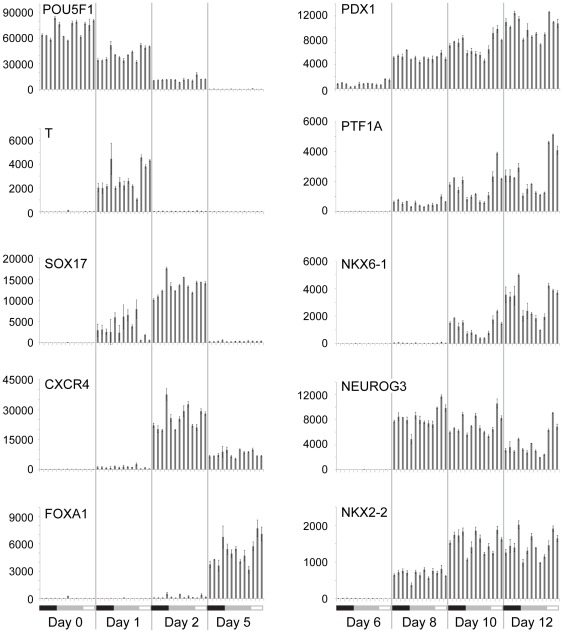
Digital mRNA profiling of scaled pancreatic differentiation runs. The dynamics of gene expression demonstrated that undifferentiated cells (POU5F1) were directed through mesendoderm (Brachyury: T), definitive endoderm (SOX17, CXCR4), primitive gut tube (FOXA1), posterior foregut (PDX1), to form pancreatic epithelium (NKX6-1, PTF1A), and endocrine cells (NEUROG3, NKX2-2). Precise temporal control and consistency between manufacturing runs indicated a reproducible and robust specification of each lineage. The plots are ordered according to CyT49 cell bank (left to right): black bar (MCB4: Expt #18–21), grey bar (RCB-Dw: Expt #25–30), open grey bar (WCB4B: Expt #35–37). The average and standard deviation of three biological replicates are plotted. Additional data is shown in Figs. S6, S7.

Gene expression analysis of the 13C group by digital mRNA profiling [24], [25] also highlighted the reproducibility of our system (Fig. 3, Figs. S6, S7), in particular the consistency of mRNA dynamics between independent experiments. In addition to the expected sequence of target mesendoderm, endoderm, gut and pancreatic lineage marker expression as cells proceed through each successive stage of differentiation, we observed the rapid downregulation of markers of undifferentiated cells, such as OCT4 (Fig. 3, POU5F1), NANOG, SOX2, DPPA4 (Fig. S6) and very low expression levels of markers for mesoderm, neural ectoderm, trophectoderm, and intestinal, or liver endoderm phenotypes (Figs. S6, S7). The precise dynamics of gene regulation from stage-to-stage and from process-to-process confirm tight control of each stage of differentiation. We expect that the elevated level of process control implemented through all phases of scaled hESC culture, aggregation and differentiation contributes to minimizing overall process variation.

### Reproducibility of Generating Functional Glucose-responsive, Insulin-secreting Cells *in vivo*

**Figure 4:**
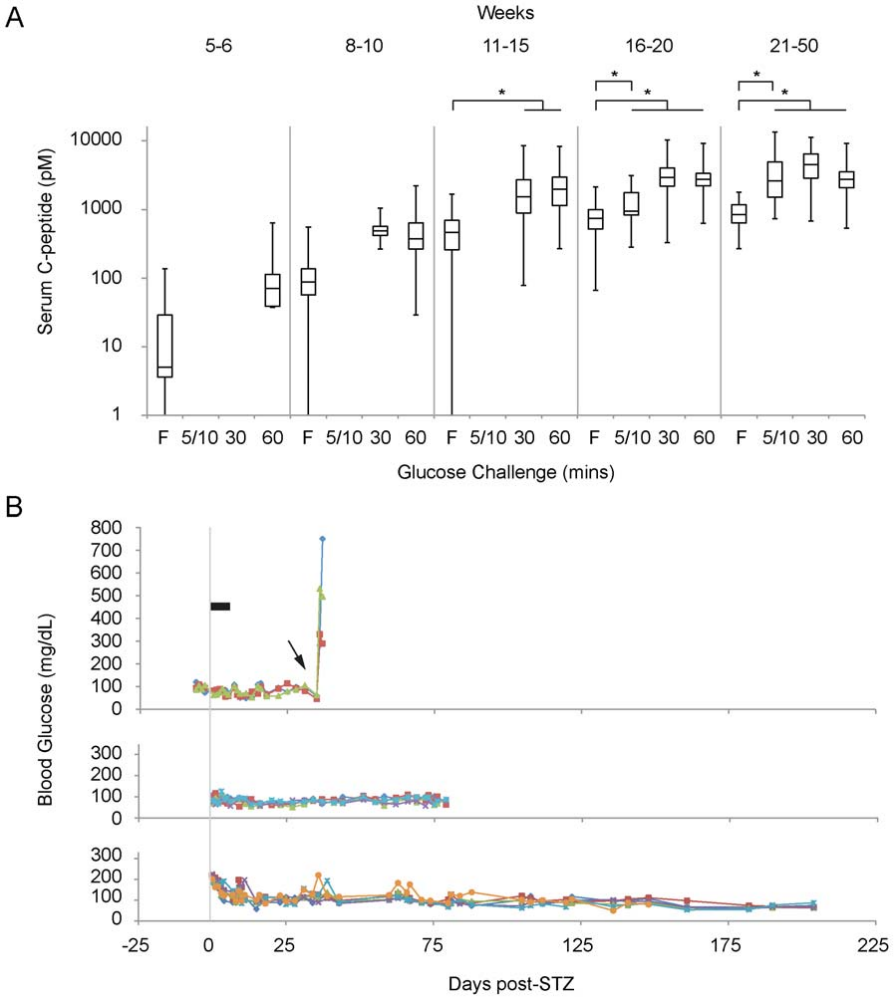
*In vivo* function of engrafted pancreatic differentiation runs. (A) GSIS response in high functioning engrafted animals (n = 166) at selected time points after implantation (weeks post-engraftment shown at top). Each time point shows F, fasting; 5 or 10 min; 30 min; and 60 min serum human C-peptide measurements. A significant increase in both fasting (p<0.01) and maximum GSIS (p<0.01) was observed from weeks 5–10 to weeks 11–50. Other statistically significant differences are indicated (*: p<0.01). The box plots show the median, second and third quartile (box), max and min values for serum human C-peptide (pM). A log scale is used to display both early and late time points. Empty plots indicate that no data were collected. Additional analyses and the number of ELISA samples for each data set are shown in Fig. S8 and Table S3, respectively. (B) Protection against STZ-mediated hyperglycemia in long-term grafts. Non-fasting blood glucose measurements (mg/dL) are plotted for three cohorts of mice (n = 15, one animal not shown) over days post-STZ treatment. STZ was administered on days 1–5 (black bar), in mice containing high-functioning grafts, 4–5 months after engraftment. Explanting grafts (arrow) led to hyperglycemia. The cohorts were derived from expt #34 (upper), #30 (middle) and #2 (bottom) (Table S2, banks RCB-D and RCB-Dw).

We tested the pancreatic populations generated in thirty-one scaled differentiation runs (Table S2) for the capacity to generate islet-like tissue with functional insulin-secreting cells after implantation into the epididymal fat pad (EFP) of male SCID/Bg mice. In total, 240 mice were implanted with typically 3×10^6^ cells, in 44 cohorts, of which 228 (95%) showed evidence of cell engraftment, remained healthy through at least 3 months and exhibited a functional response to glucose challenge (GSIS: glucose-stimulated insulin secretion, Fig. S8A). Based on our previous findings, we scored mice with a maximal-stimulated-release of >2000 pM serum human C-peptide as a high-functioning group (Figs. 4A, S8B, n = 166 mice (69%), 4574±2415 (mean ± SD) pM, weeks 21–50) which would be fully protected against STZ-induced hyperglycemia [6]. A second group of 62 mice (26%) also exhibited glucose-stimulated release of human C-peptide (Fig. S8C, 1109±573 pM, weeks 16–25), but did not reach this 2000 pM benchmark by 25 weeks post-engraftment. Such animals would be predicted to be partially protected in our STZ-lesion paradigm. To enable more refined analyses of the development of graft function, we examined the GSIS data at various time points after implantation (Fig. 4A). As we have observed previously [6], the implanted cells in the high functioning group produced increasing levels of both basal and stimulated human C-peptide as the grafts matured over time (weeks 5–10 vs. 11–50, p<0.01). Human C-peptide was detected as early as 5–6 weeks post-implant, and a statistically significant stimulation above fasting levels was first observed at 11–15 weeks. By 16–20 weeks post-implant, statistically significant GSIS was observed within 5–10 mins of glucose challenge, demonstrating maturation of the functional glucose-responsive cell mass to a state exhibiting dynamics akin to a first phase insulin response [26]. The group of partially protected mice also exhibited increasing GSIS response over time (Fig. S8C), and both groups combined exhibited an average fasting serum C-peptide of 888±356 pM and a maximum GSIS of 4430±2458 pM at 21–50 weeks (p<0.01). The 12 mice (5%) that failed to exhibit function, or were sacrificed due to poor health, were all in cohorts that otherwise exhibited high-functioning animals, suggesting a failure of engraftment rather than an inability of the implanted cells to differentiate and function *per se*. Confounding technical and health-related issues attributed to surgical complication, animal stress, fighting, or the onset of SCID-associated thymoma [6], could all manifest as poor initial engraftment, or reduced observable function. We also note that each of these thirty-one scaled differentiations, derived from 5 different banks and produced by 6 different operators, generated functional cells *in vivo* (Fig. S8), highlighting the robustness of the manufacturing system.

To examine the therapeutic potential of the implanted, functional pancreatic progenitors, we eliminated the endogenous mouse β-cells at approximately 4–5 months after engraftment. This was achieved using the β-cell toxin STZ [27] which exhibits significantly greater cytotoxic activity against murine as compared to human β-cells [28], [29]. We observed complete protection from hyperglycemia post-STZ administration in each of the 15 animals tested and this protective effect was stable for >6 months in the 6 mice maintained for that duration (Fig. 4B). Blood glucose was maintained at or below 100 mg/dL (84.4±17.3, n = 6 mice (68 bleeds), 105–204 days post-STZ), indicating continued control by human β-cells, which drive blood glucose to a lower set-point than their mouse counterparts [30]. Hyperglycemia was observed when the grafts were explanted, demonstrating that glucose homeostasis was maintained by the implanted cells and not by regeneration of mouse β-cells in the endogenous pancreas. These findings were consistent with our previous analysis of the functional capacity of mature pancreatic progenitor-derived grafts *in vivo* [6].

### Characterization of Pancreatic Grafts from Scaled Differentiation Runs

**Figure 5:**
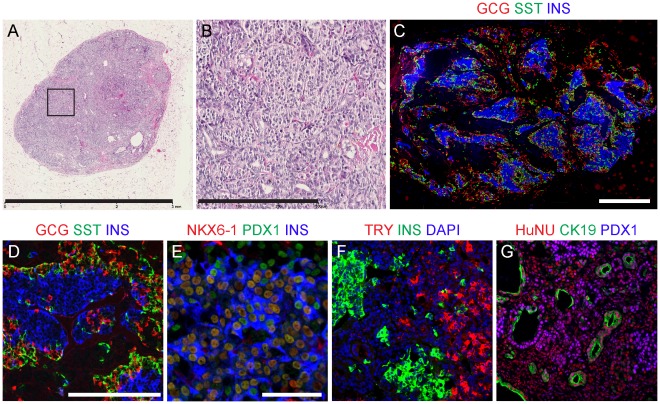
Histological and immunofluorescence analyses of CyT49-derived neo-pancreatic grafts at 18 weeks post-implant. (A) Hematoxylin and eosin staining of a graft cross-section, and (B) higher magnification of boxed area. (C, D) GCG, SST and INS staining in a cross-section of a graft, demonstrating single-pancreatic hormone expression and large clusters of INS^+^ cells. (E) Co-expression of NKX6-1, PDX1 and INS. (F) TRY, INS and DAPI staining. (G) CK19, PDX1 and HuNU staining. GCG, glucagon; SST, somatostatin; INS, insulin; TRY, trypsin; HuNU, human nuclear antigen; CK19, cytokeratin 19. Grafts were from expt #18 (A, B), and #20 (C–G). These representative mice exhibited fasting human C-peptide levels of 229–865 pM, and maximum GSIS (30 or 60 min stimulation) of 2614–3485 pM at week 15. An additional ten representative grafts are shown in Figs. S10, S11, S12, S13, S14. Scale bars: 3 mm (A), 300 µm (B), 500 µm (C), 200 µm (D, F, G), 50 µm (E).

Of the 240 mice implanted in this study, all grafts were maintained *in vivo* for at least four months, and some for greater than 6 months (n = 19 mice, 27 grafts). Measurement of grafts explanted at 16–52 weeks post-implant demonstrated that they were typically small with a greatest dimension of 5.2±2.5 mm, although in some cases grafts were larger due to the formation of cysts (mean ± SD, n = 112 mice, 191 grafts; max = 15.0 mm; min = 2.0 mm; Fig. S9). The structure and cellular composition of the implants were examined with histological and immunofluorescent analyses (Fig. 5, Figs. S10, S11, S12, S13, S14). The grafts contained large areas of endocrine clusters surrounded by mouse connective tissue and occasional small, or large, ducts with varying epithelial morphologies (Fig. 5A, B; Figs. S10, S11, S12, S13, S14). A subset of small and large grafts (n = 17) were examined independently by a board-certified pathologist at approximately 18 or 26 weeks post-implant. The pathologist characterized the grafts as pancreatic tissue comprised of islets and ducts, and noted that approximately half of these grafts were cystic (Figs. S10, S11, S12, S13, S14). These cysts were suggested to represent dilated pancreatic ducts. Immunofluorescence analyses supported the histological and physiological data. Consistent with glucose-stimulated insulin secretion, the grafts exhibited large clusters of insulin^+^ cells with PDX1^+^ and NKX6-1^+^ nuclei (Fig. 5C–E). Cells immunoreactive for other pancreatic hormones, including glucagon, somatostatin, ghrelin, and pancreatic polypeptide were also observed (Fig. 5C, D; data not shown). The grafts also occasionally displayed trypsin^+^ exocrine cells (Fig. 5F) and cytokeratin 19^+^/PDX1^+^ ductal cells (Fig. 5G), demonstrating the potential to generate all three pancreatic lineages *in vivo*, as described previously [6], [7]. These cell components were confirmed to be derived from the implanted cells as determined by staining for a human nuclear specific antigen (Fig. 5G, data not shown). The histological and immunofluorescent analyses therefore indicated that the grafts were predominantly comprised of endodermal derivatives, principally pancreatic tissues.

## Discussion

**Figure 6:**
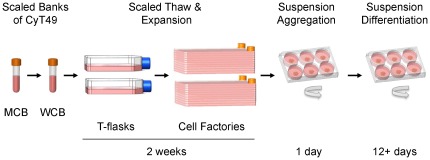
Schematic representation of the manufacturing process for pancreatic progenitors. Scaled and high-density single-cell master (MCB) and working cell banks (WCB) of CyT49 cells were prepared with cGMP and serve as a virtually unlimited source of starting material for differentiation. Cryopreserved vials of 10^7^ CyT49 cells from a qualified working cell bank are thawed and expanded in adherent culture conditions for 4 or 5 passages over a 2-week period. A single cell suspension is harvested and aggregated in rotational culture in 6-well trays. After 24 hrs the hESC aggregates are differentiated en masse with the 4-stage protocol to a population of pancreatic progenitors. For clinical development, scaled lots of differentiated aggregates will be produced with cGMP and cryopreserved, enabling a proportion of each lot to be tested for safety, efficacy and other regulatory considerations. Qualified lots of differentiated pancreatic progenitors will be thawed, recovered, formulated, loaded into a durable immunoisolation device and delivered for preclinical or clinical studies. The manufacturing process is amenable to further scaling via additional passages in adherent culture, as well as aggregation and differentiation in larger vessels.

We report here an integrated manufacturing process for a hESC-based therapeutic candidate for type 1 diabetes using the CyT49 cell line (Fig. 6). High-density, single cell banks of CyT49 were thawed and expanded with efficient population doublings. Expanded cultures were aggregated in suspension, generating uniform clusters of undifferentiated cells, which were then differentiated in suspension en masse. An optimized 4-stage protocol directed the stepwise formation of highly enriched pancreatic populations that functioned robustly *in vivo*. The process integrates a standardized cell source and scaled differentiation with the ability to cryopreserve the end-stage pancreatic aggregates so that function is retained *in vivo* (not shown). This provides the critical ability to test function and safety of scaled manufactured lots, prior to pre-clinical studies or clinical application. Our approach represents the first demonstration of a practical system for manufacturing a hESC-based treatment for type 1 diabetes, mitigating many of the perceived hurdles to clinical development.

In contrast to previous reports of cGMP banking of clinically-relevant hESC lines, which by necessity relied on earlier clump-passaging methodologies [31], our single-cell suspension cGMP MCB/WCB are a starting point for acute scaled expansion for producing large batches of pancreatic progenitors. WCB4B vials could theoretically be thawed on a monthly basis, generating batches of >10^10^ cells each month, and at that rate would provide starting material for more than 20 years of manufacturing. If exhausted, additional WCBs derived from MCB4 could be generated as required. Our strategy also minimizes the potential for karyotypic drift in euploid hESC cultures [17], [32], as banked cells are only expanded for 3–6 passages over a 15–20 day period before differentiation. It is notable that while theoretical considerations implicate population size as a risk factor in accumulating aneuploidies in hESC cultures [33], our conditions could support the expansion of euploid cells on a clinical-manufacturing scale. The simplicity of this expansion system for CyT49 is also likely to be amenable to automation.

Aggregation of undifferentiated hESC in dynamic rotational suspension culture is a unique methodology. Current examples of suspension culture of hESC use aggregates generated from collagenase passaging [34], shear of suspension clusters [35], in static culture conditions [34], require microcarrier support [36], [37], [38], [39], or heat shock in combination with the ROCK inhibitor Y27632 to support aggregation and survival [40]. Such limitations add hurdles to maintaining cells in an undifferentiated state, the ability to scale and automate, or support effective population expansion in the face of passaging inefficiencies. Conversely, we have demonstrated that uniform clusters of undifferentiated hESC could be aggregated with high incorporation efficiencies in rotational culture, by taking advantage of the inherent self-associative properties of hESC. The particular conditions we optimized were critical for effective aggregation, as the circular movement imposed a shallow central vortex drawing cells into a high local density in the middle of each well. The epithelial characteristics of undifferentiated hESC, presumably E-cadherin expression in particular [41], may mediate effective self-association of cells as they collide [42]. Aggregates did not form efficiently in static suspension culture, rocked, or stirred cultures, or even in centrifuged cell pellets (not shown), indicating that fluid movement and limited shear forces played an important role in transitioning cell-cell contact into stable adhesion, a well characterized phenomenon [43]. The ability to serially passage aggregates of euploid hESC in dynamic rotational culture suggests that future manufacturing processes may also utilize scaling of hESC cultures in such a format.

The pairing of scaled expansion of CyT49 in adherent culture with aggregation and differentiation en masse is also a novel strategy. Effective step-wise lineage specification en route to pancreatic cell types was achieved by adapting our previous adherent-based system to dynamic suspension culture. This approach is essentially unrelated to traditional embryoid body differentiation, which is stochastic at best and not capable of directing uniform specification. Our strategy is also conceptually different from efforts to expand endoderm intermediates such as DE [11]. In the present method, differentiation was directed in a highly controlled manner and resulted in consistency and uniformity of cellular composition superior to that we reported previously. In contrast to differentiation of hESC in adherent culture, which in our hands appears to be sensitive to variations in local densities, hESC aggregates by their very nature have high and uniform local cellular density, which may exert a positive influence on the uniformity of pancreatic differentiation. We demonstrated with our previous methods that multiple hESC lines could be differentiated to pancreatic lineages [5], [6], [7], including CyT203-derived glucose responsive, insulin secreting cells *in vivo* [6]. Furthermore, others have recently generated functional grafts from the WA1 hESC line [44]. Given the likely requirement for cell line-specific optimization of culture conditions and timing [5], [6], it is reasonable to expect that these suspension methodologies could be applied to other pluripotent cell lines. The mechano-physical properties of the cellular microenvironment are also likely to be quite different in suspension as compared to adherent conditions, which may also contribute to consistency of cell fate determination. We have achieved defined cell compositions without the requirement for cell sorting and the associated poor yields that accompany it [7], although sorting of the dissociated Stage-4 aggregates to enrich PE or endocrine cells for profiling analyses was achieved using CD142 and CD200, respectively (data not shown). Furthermore, for a cellular therapy based on implanting pancreatic lineages, the use of cellular aggregates offers a significant advantage over microcarrier-based suspension technologies. Pancreatic aggregates can be implanted without disrupting the maturing cellular architecture, avoiding substantial losses that would occur when harvesting from microcarriers.

The manufacturing process we have developed serves as a foundation for additional scaling, development of conditions for cGMP manufacturing and production of qualified material for preclinical and clinical studies. The Edmonton protocol calls for a patient dose of 10,000 islet equivalents (IEQ)/kg body weight to achieve the primary endpoint of insulin independence [45]. A projected dose suggests that a large number of hESC-derived pancreatic progenitors will be required for clinical application, estimated to be a minimum of 10^8^ cells/patient [7]. A sensitivity analysis of the scale required to enter a phase 1 clinical trial needs to account for the number of patients, absolute doses to be tested, amount of product utilized in quality control testing, and efficiencies at each step of the manufacturing process (Fig. 6). Given the assumptions that can be made for each variable and allowing for the range within these may fall, we can speculate that a batch of somewhere between 2.5×10^9^ and 3.3×10^10^ CyT49 cells will be necessary to generate sufficient cell product for a phase 1 clinical trial of ten patients at a dose of 10^8^ cells/patient. In this report we have demonstrated the ability to reach the lower end of this predictive window using our current technology. A single vial of 10^7^ cells was thawed, expanded over two weeks, and differentiated to produce pancreatic aggregates of 3.3×10^9^ cells, which functioned appropriately *in vivo* (data not shown). The upper end of this prediction is also well within reach, as additional passages in multilayer chambers are neither technically difficult nor approaching the limit of the technology. A single 40-stack cell factory has a surface area of 25,000 cm^2^, which conservatively would yield >6.2×10^9^ CyT49 cells under our present conditions. Given the progress in automating hESC expansion with robotics, the application of hESC expansion in suspension culture, or the logical adaptation of differentiation to controlled, expandable, and closed bioreactor manufacturing systems, we anticipate that we will be able to increase the scale of our process by several additional orders of magnitude.

Assessing the consistency that could be achieved with a scalable manufacturing process for hESC-derived pancreatic progenitors was the primary objective of this study. CyT49 could be differentiated with high reproducibility using an optimized process, achieving robust function *in vivo*. In concordance with our previous reports, we generated pancreatic grafts that could sense blood glucose and respond by releasing human insulin [6], [7]. The grafts differentiated and matured over time, as shown by the statistically significant increase in baseline and amplitude of response observed after week 10, and significant 5 to 10 min GSIS response at weeks 16–50. Critically, grafts could maintain blood glucose homeostasis in an endogenous β-cell ablation model. The combination of scaled differentiation and functional outcome in hundreds of animals represents an experimental magnitude far greater than previously reported, with reproducibility that enables progression to formal preclinical development. We envision developing an allo-compatible neo-pancreatic product, by engrafting pancreatic progenitors within a vascularizing and durable immunoisolation, or macroencapsulation, device.

Suspension-based pancreatic differentiation runs consistently yielded only minimal amounts of non-pancreatic tissue upon implantation, in contrast with the variability in teratoma rates displayed in our previous reports [6], [7]. Nonetheless, some grafts also contained dilated ducts and/or cysts derived from these ducts. While not a particular safety concern pathologically, an enlarged cyst could potentially impinge upon surrounding tissue. Cell implantation within a durable macroencapsulation device could potentially constrain such structures, and offer an additional level of safety by enabling retrievability of implanted cells. In any format, formal demonstration of product safety requires both regulated preclinical studies, and eventual batch release qualification of cryopreserved material produced under cGMP.

In summary, we have assembled and demonstrated the utility of a system for the manufacturing of a functional hESC-based therapeutic product for type 1 diabetes. Our approach coordinates many discrete steps into a highly regulated process, linking a scaled and standardized cell source with expansion and scalable differentiation, through to qualification *in vivo*. The process generates implantable material with reproducibility and the compatibility required for industrialization.

## Materials and Methods

### Ethics Statement

Animal experiments were performed at Absorption Systems, San Diego, CA and were approved by their Institutional Animal Care and Use Committee, protocol #VC-02-09-148.

### Undifferentiated hESC Culture

The CyT49 hESC line ([6], NIH registration number: 0041) was used in these studies. Xeno-free growth media (XF HA) consisted of DMEM/F12 containing GlutaMAX (Life Technologies, cat#10565) supplemented with 10% (v/v) of Xeno-free KnockOut Serum Replacement (Life Technologies, cat#12618-001), 1% (v/v) non-essential amino acids (Life Technologies, cat#11140-050), 0.1 mM 2-mercaptoethanol (Life Technologies, cat#21985-023), 1% (v/v) penicillin/streptomycin (Life Technologies, cat#15070-063), 10 ng/mL heregulin-1β (Peprotech, cat#100-03) and 10 ng/mL activin A (R&D Systems, cat#338-AC). Upon thaw, or at regular passaging, dissociated hESC were plated at 50,000 or 33,000 cells/cm^2^ for three and four day growth cycles, respectively. On the day of plating only, cell attachment was facilitated by including 10% (v/v) of non-heat inactivated human AB serum (Valley Biomedical, cat#HP1022). A standardized plating volume of 0.2 mL/cm^2^ was used for different tissue culture plates, T-flasks and cell factories. The volume of growth media used was increased for each additional day of feeding according to Methods S1, and did not include human serum. On the day of passaging, cultures were fed with fresh growth media and cultured 4–8 hrs before dissociation. Cultures were washed with PBS (Life Technologies, cat#10010-031) and dissociated for 6 mins at 37°C using pre-warmed Accutase (Innovative Cell Technologies, cat# AT104). Dissociated cells were gently collected using 3× volume of cold hESC media (without heregulin or activin), counted using a ViCell automated cell counter (BD Biosciences), or a hemocytometer, centrifuged for 5 mins at 200× g and the pellet resuspended in fresh growth media at 1–10×10^6^ cells/mL for subsequent plating. Where indicated, the StemPro hESC SFM medium (Life Technologies, cat#A1000701) supplemented with 10 ng/mL heregulin-1β, 10 ng/mL activin A, 10 ng/mL FGF2 and 200 ng/mL LR^3^-IGF1 (StemPro), or the same media without FGF2 (SP HAI) were used for hESC culture or suspension culture. Cell culture was performed in humidified incubators at 37°C and 8% CO_2_.

### Cryopreservation and Banking of hESC

Small and large-scale single-cell banks were cryopreserved using the same approach, essentially as described previously [46]. Adherent cell cultures were harvested according to the above passaging protocol, pooled and counted. Cell pellets were resuspended in prewarmed 50% hESC culture medium (without growth factors)/50% human serum. An equal volume of 80% hESC culture medium (without growth factors)/20% DMSO was added drop-wise, with swirling. 1 mL of cells was distributed to 1.8 mL Nunc cryovials for freezing at −80°C in Nalgene Mr Frosty containers for 24 hrs, before transferring to liquid N_2_. cGMP culture and banking were performed by Viacyte employees at a certified 3^rd^ party contract research organization.

### Aggregate Formation and Differentiation

Scaled pancreatic differentiation runs in suspension typically utilized 18–20 6-well trays and were carried out by first generating hESC aggregates. On the fourth day after passage adherent hESC cultures were fed with fresh XF HA media and cultured 4–8 hrs before dissociation. Cultures were harvested according to the passaging protocol and resuspended in approximately 10 mL cold XF HA media per 150 cm^2^ culture surface area. Cells were counted using a ViCell automated cell counter, then seeded into ultra-low adhesion 6-well trays (Greiner BioOne, cat#657185) at 1×10^6^ cells/mL in XF HA media and 5.5 mL per well. The 6-well trays were incubated at 37°C on orbital rotators set at 95 rpm (Innova2000, New Brunswick Scientific). During overnight culture, single cells aggregated to form spherical clusters approximately 100–200 µm in diameter (Figs. S4, S5) and roughly 5,000 aggregates per well. The differentiation media and rotation conditions used for each day are detailed in Methods S1. To initiate differentiation, aggregates were pooled into conical tube(s) and allowed to settle by gravity, or in some experiments by centrifugation at 500 rpm for 90 seconds, followed by a wash using Stage-1 media without growth factors. The aggregates were re-settled, then resuspended in day 1 media and evenly redistributed to the same number of wells using new 6-well trays in 5.5 mL total volume per well. After 20–24 hrs of differentiation some cell death and a wispy mass, or clump, of DNA was visible in each well (Fig. S5A). On this day, clumps were disrupted by triturating with a P200 pipettor and returned to the rotator for 30 seconds to allow aggregates to collect in the center of the well. Approximately 4.5 mL of media was aspirated from each well and wells were fed with 4.5 mL of day 2 media. Aspiration of 4.5 mL of media followed by feeding with 4.5 mL of the subsequent day’s media was performed daily thereafter. In some experiments, at the end of Stage-2 the aggregates were pooled and redistributed to half the original number of wells in 5.5 mL of day 5 media. Aggregates were sampled at the indicated time points for histological, immunofluorescence, and gene expression analyses.

### Digital Gene Expression Analysis

Total RNA was isolated from triplicate samples using the 6100 nucleic acid extractor (Applied Biosystems; Foster City, CA) and 100 ng used per reaction for quantitation of gene expression using the nCounter System [25] according to the manufacturer’s instructions (Nanostring; Seattle, WA). The raw count data were normalized to the count data from internal control sequences (“spikes”). After spike-normalization, the data were normalized to the count data from four different housekeeping genes using geometric means of the spike-normalized counts for ACTB, POLR2A, PPIG, and TBP. The average and standard deviations of the fully normalized counts were calculated for three biological replicates.

### Flow Cytometry Analysis

Flow cytometric analyses were conducted essentially as described previously [7]. Undifferentiated hESC, hESC-derived DE aggregates, or hESC-derived pancreatic aggregates, were washed in PBS and then enzymatically dissociated to single cell suspensions at 37°C using Accutase (hESC), or Accumax (aggregates) (Innovative Cell Technologies). MACS Separation Buffer (Miltenyi Biotec #130-091-221) was added and the suspension was passed through a 40–100 µm filter and pelleted. For surface marker staining, dissociated cells were incubated with fluorescent-conjugated antibody diluted in MACS Separation Buffer for 20 mins and then washed in MACS Separation Buffer. Following dissociation, and in some cases surface marker staining, cells were fixed for 30 mins in 4% (wt/v) paraformaldehyde, washed in FACS Buffer (PBS, 0.1% (wt/v) BSA, 0.1% (wt/v) NaN_3_) and stored in IC Buffer (PBS, 1% (wt/v) BSA, 0.1% (wt/v) NaN_3_). For intracellular staining cells were permeabilized with Perm Buffer (PBS, 0.2% (v/v) Triton X-100, 5% (v/v) normal donkey serum, 0.1% (wt/v) NaN_3_) for 30 mins on ice and then washed with IC buffer. Cells were incubated with primary antibodies diluted with Block Buffer (PBS, 0.1% (v/v) Triton X-100, 5% (v/v) normal donkey serum, 0.1% (wt/v) NaN_3_) overnight at 4°C. Cells were washed in IC buffer and then incubated with appropriate secondary antibodies for 60 mins at 4°C. Cells were washed with IC Buffer and then in FACS Buffer. Cells were resuspended in FACS buffer for flow acquisition. Antibodies and dilutions used for staining are detailed in Methods S1. Flow cytometry data were acquired as described previously [7] with a FACSCalibur™ (BD Biosciences), using excitation lines at 488 nm and 635 nm and detecting fluorescence at 530±15 nm, 585±21 nm, and 661±8 nm. Data were analyzed using FlowJo software (Tree Star, Inc.). Intact cells were identified based on forward (low angle) and side (orthogonal, 90°) light scatter. To account for the varying levels of autofluorescence observed in these complex samples, additional gating of intact cells (2–3 sub-populations) was performed and cells were subsequently analyzed for fluorescence intensity. Background was estimated using unstained, secondary-antibody alone, or irrelevant antibody controls, as appropriate. In figures, a representative flow cytometry plot is shown from one of the sub-populations. Numbers reported on plots and in tables represent the percentage of total cells from the intact cells gate.

### Graft Preparation and Implantation

Pancreatic populations were grafted to the EFP as described previously [6]. Most aggregates were collected and prepared at day 12 of differentiation, while some were maintained in Stage-4 medium until d14 or d16 before preparation. Briefly, the aggregates were allowed to settle by gravity and 5 µL of cell aggregate slurry (representing 1.5×10^6^ cells) was transferred onto 6 mm diameter by 0.8–1.0 mm thick gelatin foam disks (Vetspon®. Novartis Animal Health US, Inc, cat#96001) that were prewetted with RPMI 1640 with 2% (v/v) FBS. The implantable constructs were transferred to culture dishes with pre-warmed medium until implantation, typically the same day. For implantation surgery, male SCID/Bg mice (Harlan) 6–13 wks of age were anesthetized by intra-peritoneal injection of an anesthetic cocktail (90 mg/kg ketamine and 10 mg/kg of xylazine) and abdomens were shaved and swabbed with povidone iodine solution. A 1-cm midline incision was made through both skin and abdominal muscle layers, and one of the EFP was carefully externalized through the incision and placed on a saline-dampened gauze pad. A single implant construct was wrapped in the EFP and fixed in place with a small amount of veterinary tissue adhesive (VetBond, 3 M, cat#1469SB) and the EFP was returned to the abdominal cavity. The procedure was typically repeated on the contralateral EFP, for a total of two implant constructs per mouse. Absorbable suture were used to close the abdominal wall and wound clips were used to close the skin incision.

### Glucose Stimulated Insulin Secretion (GSIS) Assays

Starting from 35 days, but typically at about 42–56 days post-implantation, graft function was assessed by performing measurement of serum human C-peptide in response to glucose administration. Prior to the GSIS assay, mice were fasted for approximately 15–18 hrs. Glucose was administered via intra-peritoneal injection of a 30% dextrose solution at a dose of 3.0 g/kg body weight, and blood was collected prior to (fasting) and at combinations of 5, 10, 30, or 60 mins after glucose administration. Blood samples of approximately 20–40 µL were collected by retro-orbital sinus puncture and transferred to microtainer tubes (BD Biosciences, cat#365956) containing blood/serum separation gel. Serum was collected after spinning these tubes at 1000–2000× g for 5–10 mins. The ELISA used for human C-peptide (Mercodia Ultrasensitive Human C-peptide ELISA, cat#10-1141-01) was the same as used previously [6], [7]. We regard the lower limit of accurate quantitation of human C-peptide in serum samples to be 50 pM [6]. The level of cross-detection of mouse C-peptide is <1%, or a maximum of 30 pM in glucose-stimulated control mice. The level of cross-detection of human insulin is <0.0006% and <1.8% for proinsulin.

### Streptozotocin (STZ) Treatment of Mice

Mice received 70 mg STZ (Sigma-Aldrich, cat#S0130) per kg body weight, through intra-peritoneal injection on five consecutive days for a total dose of 350 mg/kg as described previously [6]. Under these conditions non-implanted animals typically reach blood glucose levels greater than 300 mg/dL, indicating depletion of endogenous β-cells [6]. For blood glucose measurements, tail vein samples were tested using glucometers specifically calibrated for measuring rodent blood glucose values (Alpha-Trak glucometer and Omnistrips, Abbott Animal Health #32004-05 and #32006-05).

### Statistical Analyses

One-way ANOVA was performed for all statistical analyses using the StatPlus package for Excel (AnalystSoft, Alexandria, VA), with p<0.01 considered significant.

### Histopathological Analysis

Grafts from mice were explanted and fixed in 10% neutral buffered formalin for paraffin embedded blocks, and in 4% (wt/v) paraformaldehyde for 4 hrs in 4°C for frozen blocks, followed by washing with PBS for 3 hrs and then equilibration in 30% sucrose at 4°C overnight. Samples of cell aggregates were made into frozen blocks by fixing with 4% (wt/v) paraformaldehyde for 30 mins and then with 30% sucrose at 4°C overnight. The paraffin embedded specimens were sectioned at 5 µm and OCT compound mounted frozen blocks were sectioned at 8 µm. Standard histological hematoxylin and eosin (H&E) staining were performed on both graft and cell aggregates. The H&E images were captured by a Nanozoomer digital slide scanner (Olympus Inc.). Immunofluorescence analyses of aggregates and grafts were performed as described previously [5], [6], [7], [41]. The antibodies used are detailed in Methods S1. Images were acquired with a confocal microscope (Nikon, Eclipse 80i, Ci).

## Supporting Information

Figure S1 **Scalable feeder-free culture system for CyT49 expansion.** (A) Phase contrast images of single-cell passaged CyT49 at six time points after plating. Single plated cells (3 hr) migrate to form micro-colonies (d1), which proliferate to form a near confluent monolayer of hESC (d5). (B) Cell counts of serially passaged CyT49 cells in xeno-free culture media confirmed that addition of FGF2 was not required for long-term self-renewal under these conditions (XF HA vs XF HAF). A control condition (SR AF HS), which also supports self-renewal of undifferentiated hESC (data not shown) was included. The base media was DMEM/F12, and the cultures were passaged with Accutase and maintained in 6-well trays. XF: 10% xeno-free serum replacer, SR: 20% knockout serum replacer, H: 10 ng/mL heregulin-1β, A: 10 ng/mL Activin A, F: 10 ng/mL FGF2, HS: 10% soluble-phase human serum for attachment (first day of each passage only). (C) CyT49 maintained in XF HA for 10 passages retained uniform expression of OCT4. DAPI, 4′,6-diamidino-2-phenylindole. Scale bars: 100 µm.
(TIF)

Figure S2 **Optimization of the feeder-free culture system for scaled expansion of CyT49.** (A) Determination of the cell yield 1–6 days after plating with three different densities (0.66, 1.0, or 1.5×10^6^ cells/60 mm dish, or 3.3, 5.0, or 7.6×10^4^ cells/cm^2^, respectively) demonstrated that near-exponential expansion occurred for the first four days. In comparison to a plot of theoretical-low expansion (plating 0.5×10^6^ cells/60 mm dish, 24 hr population doubling), the cell yield did not continue to increase after day four. Slowing of expansion was detected earliest, and was most prominent at, the highest plating density, suggesting that culture confluence may restrict further yield increases. These studies contributed to electing to use densities of 5×10^4^ cells/cm^2^ for a 3-day culture interval, and 3.3×10^4^ cells/cm^2^ for a 4-day culture interval during large-scale processes. (B) Pre-feeding of cultures prior to passaging improves plating efficiency. Cultures were fed with fresh media for 1, 2, 4, or 8 hrs prior to passaging (n = 2 dishes per time point). A moderate increase in cell yield was observed with pre-feeding (grey columns, left axis), as well as in plating efficiency (black bars, right axis). Plating efficiencies were determined by counting cultures 24 hrs post-plating. (C) The duration of Accutase dissociation was standardized to a 6-minute exposure by determining that it supported effective plating efficiencies for both pre-fed and non pre-fed cultures compared to a 4-, or 8-minute treatment. (D) In order to reduce the number of handling steps in scaled-expansion processes, we demonstrated that Accutase could still disaggregate cultures effectively if it was added, then immediately aspirated from vessels. The residual activity (“aspirated accutase”) was sufficient to disaggregate cultures and provide a plating efficiency of >85%. The plating efficiency of cultures grown in 60 mm dishes, pre-fed for 7 hrs, and then exposed to aspirated Accutase for 4, 6, 8, or 10 minutes are shown, as is a control plate passaged with standard Accutase treatment for 6-minutes (Cont.).
(TIF)

Figure S3 **CyT49 cell banks.** (A) Derivation and genealogy of CyT49 cell banks. Stepped lines indicate a thaw and expansion from the indicated bank. cGMP conditions indicated by the dashed box. (B) Phase contrast images of thawed CyT49 cultures from the RCB-Dw and WCB4B large-scale cell banks, imaged with a 20× objective at day 1 or 3 (left, center panels. Scale bar: 200 µm), or with a 40× objective at day 3 (right panels. Scale bar: 50 µm). Thawed cultures exhibited a primarily undifferentiated morphology. (C) Immunofluorescence analysis of cultures one passage after thaw, demonstrating maintenance of expression of OCT4 and NANOG. Scale bar: 100 µm. (D) Flow cytometric analysis of thawed cultures from RCB-Dw, MCB3, MCB4 and MCB5, co-stained with anti-CDX2, anti-OCT4, and anti-SSEA4. The analyses were first gated on the CDX2^dim^ population (upper), followed by plotting OCT4/SSEA4 co-positive cells (lower). The population-wide (total) percentage of gated cells is shown. Typically, >98% of the population was comprised of CDX2^dim^/OCT4^+^/SSEA4^+^ undifferentiated cells. SSC: side scatter. Methods for labeling and cytometry were essentially as described previously [7].
(TIF)

Figure S4 **Aggregation and serial culture of hESC in suspension as undifferentiated aggregates.** (A) Single cell suspension of CyT49 after 4 hrs of aggregation with rotational culture showing initial cluster formation, and (B) aggregates after 24 hrs in XF HA media. The BG02 hESC line was also used in some of these studies [47]. (C) BG02 aggregates after 2 days culture in StemPro medium. (D) Incorporation efficiency and expansion of CyT49 in aggregates in XF HA or SP HAI medium (left axis: columns  =  cell counts; right axis: diamond and grey bar  = % of input cells). Cells were aggregated in triplicate, in 6-well trays, in 5.5 mL at 10^6^ cells/mL (seeded). The incorporation efficiencies were estimated after 24 hrs by two methods. First, counting the number of live unincorporated cells and extrapolating, indicated that 72±3.2% and 76±1.5% of input cells were incorporated (% Inc.) for XF HA and SP HAI conditions, respectively (mean ± SD, n = 3). Secondly, cell counts of dissociated aggregates (d1) indicated that they contained 63.5±2.2% and 98.9±10.1% of input cells for XF HA and SP HAI conditions, respectively. After 3 days of expansion (d3) aggregates contained 162.4±14.7% and 248.7±12.7% of input cells for XF HA and SP HAI conditions, respectively. (E) Diameters of undifferentiated CyT49 aggregates. Aggregates were imaged under 3× magnification on a dissecting microscope and diameters were measured with the ImagePro software. Triplicate wells were imaged and 100 aggregates were measured for each image (n = 3×100). The box plots show the median, second and third quartile (box), max and min values for each condition. (F) Representative SP HAI aggregates from (D,E), and (G) XF HA aggregates from (D,E), imaged with an inverted microscope. (H) Hematoxylin and eosin and (I) anti-OCT4 stained sections of BG02 aggregates grown in StemPro medium demonstrating undifferentiated morphology, lack of overt differentiation, layer formation or cavitation, and uniform expression of OCT4. (J) Western blotting demonstrated that E-cadherin protein was degraded by Accutase passaging (Acc. 0 hr) compared to EDTA-based dissociation (Versene, Ver 0 hr), but was rapidly re-expressed during the first 3–6 hrs of aggregation (XF HA conditions). Markers shown in kD. (K–N) The ability to maintain hESC in suspension culture was demonstrated by serially passaging CyT49 aggregates in XF HA media for 10 passages. Cultures were passaged by dissociating aggregates to single cells, and re-aggregating in rotational culture. No spontaneous differentiation was observed. Cells were subsequently plated in adherent culture for immunofluorescence analysis, and exhibited uniform expression of (K) OCT4 and (L) TRA-1-81 after 10 passages (∼2 months) in suspension culture. Matched 4′,6-diamidino-2-phenylindole (DAPI) staining images are shown in the right panels in greyscale. (M) These cultures had also retained a normal karyotype (46,XY [20/20 metaphase spreads]). (N) qPCR analysis of CyT49 cells cultured in adherent XF HA conditions, or in suspension culture (Susp p6) for >1 month (6 serial passages) in the defined media described previously [18] containing 200 ng/mL LR^3^-IGF1, 10 ng/mL HRG and 10 ng/mL ActA. Suspension aggregates retained robust expresson of markers of pluriptency (POU5F1/OCT4, NANOG and SOX2), but did not express appreciable levels of markers upregulated during differentiation (Eomes, MIXL1, CXCR4, SOX17, HNF1B, HNF4A). Differentiating aggregates at Stage-1 (d2) and Stage-2 (d5) were included as controls. Fold values are plotted relative to the XF HA sample. These data suggest that hESC could be incorporated efficiently into aggregates, expanded, and serially-passaged as euploid undifferentiated cells. Similar results were observed using BG02 (not shown). Suspension aggregates could also be differentiatiated to representatives of all three germ layers in teratomas in immunocompromised mice (not shown). Scale bars: 200 µm (A–C), 100 µm (F,G), 50 µm (H,I,K,L).
(TIF)

Figure S5 **Characterization of pancreatic differentiation in rotational suspension culture.** (A) Low magnification (dissecting microsope 1× objective) and phase contrast (10× objective) imaging of undifferentiated aggregates (d0) and differentiating aggregates at d1, 2, 5, 8 and 12. Some large-scale differentiations, including this example, were pooled at the start of Stage-3, consolidating to half as many total wells. (B) Box plots of diameter measurements of six independent scaled differentiation runs (left to right: Table S2, Expt #4, 9, 10, 21, 27, 32) show the median, second and third quartile (box), max and min values for each data set (left axis). The mean ± SEM for the full data is shown (black squares) on the right axis (µm), with the scale shifted for clarity. (C) Additional examples of H&E staining of sectioned aggregates at d5 and (D) d12 of differentiation. Scale bars: 300 µm. (E,F) Additional examples of immunofluorescence analysis of sectioned d12 differentiated aggregates. (E) Expression of PDX1, NKX6-1 and CHGA; (F) expression of PDX1, FOXA2 and NKX2-2.
(TIF)

Figure S6 **Digital mRNA profiling of Stages-1 and -2 of scaled pancreatic differentiation runs.** Markers are displayed as groups depicting undifferentiated cells (ES), mesendoderm (ME), definitive endoderm (DE), primitive gut tube (PG), and non-pancreatic off target probes for the undifferentiated aggregates (d0) and the early stages of differentiation (d1, 2, 5). The plots of the C13 group are ordered according to CyT49 cell bank (left to right): black bar (MCB4: Expt #18–21), grey bar (RCB–Dw: Expt #25–30), open grey bar (WCB4B: Expt #35–37). The average and standard deviation of three biological replicates are plotted.
(TIF)

Figure S7 **Digital mRNA profiling of Stages-3 and -4 of scaled pancreatic differentiation runs.** Markers are displayed as groups depicting primitive gut/pancreatic (PG/pancreatic), posterior foregut (PF), pancreatic endoderm (PE), endocrine progenitor (EP), endocrine, and non-pancreatic off target probes for the later stages of differentiation (d6, 8, 10, 12). The plots of the C13 group are ordered according to CyT49 cell bank (left to right): black bar (MCB4: Expt #18–21), grey bar (RCB-Dw: Expt #25–30), open grey bar (WCB4B: Expt #35–37). The average and standard deviation of three biological replicates are plotted.
(TIF)

Figure S8 ***In vivo*** **function of engrafted pancreatic differentiations.** GSIS response in (A) all functioning engrafted animals (n = 228), (B) high functioning engrafted animals (n = 166 mice), (C) the “partially protected” group (n = 62), and the high functioning animals split into their respective starting CyT49 banks: (D) RCB-D (n = 75), (E) RCB-Dw (n = 37), (F) MCB3 (n = 17), (G) MCB4 (n = 23) and (H) MCB5 (n = 15). The data are presented as selected weeks post-engraftment. Each group shows fasting (F), 5 or 10 minute (5/10), 30 minute, and 60 minute serum human C-peptide measurements. The box plots show the median, second and third quartile (box), max and min values for serum human C-peptide (pM). Empty plots indicate that no data were collected. The number of ELISA samples for each data set is indicated in Table S3.
(TIF)

Figure S9 **Histological and size analysis of CyT49-derived neo-pancreatic grafts.** Images of explanted grafts and matching H&E stained sections: (A) left and (B) right grafts from one mouse, 25 weeks post-transplant, derived from expt #18 (MCB3). (C) 26 weeks post-transplant graft from expt #3 (RCB-D). The boxed regions are magnified in the adjacent columns (in left and right order). The week-23 fasting serum C-peptide levels, 30 min and 60 min GSIS stimulation for these representative mice are indicated. Scale in left column indicated by mm ruler. Scale bars for H&E panels: left column (A,B) 3 mm, (C) 6 mm; right two columns 300 µm. (D) A total of 191 explanted grafts from 112 mice (145 grafts/84 mice from the high functioning group; 46 grafts/28 mice from the partially protected group) were measured prior to histological processing and were grouped according to weeks post-implantation: Weeks 16–21 (n = 51 mice, 95 grafts); Weeks 22–27 (n = 45 mice, 75 grafts); Weeks 34–52 (n = 16 mice, 21 grafts). The average and standard deviation of the longest axis was plotted. Total: 5.2 mm±2.5 mm; max = 15.0 mm; min = 2.0 mm.
(TIF)

Figure S10 **Histological and immunofluorescence analysis of CyT49-derived neo-pancreatic grafts.** Two representative functioning grafts (A1, A2) are shown from expt #20 (bank MCB4, Table S2), with matching left and right EFP grafts from one mouse. Hematoxylin and eosin staining of a graft cross-section, a composite graft-wide image of glucagon (red), somatostatin (green) and insulin (blue) expression, and higher magnification of the boxed region(s) are shown for each graft (in upper and lower order). The fasting serum C-peptide levels, 30 min and 60 min GSIS stimulation for this mouse at week 15 are indicated. Scale bars (H&E image): 3 mm.
(TIF)

Figure S11 **Histological and immunofluorescence analysis of CyT49-derived neo-pancreatic grafts.** Two representative functioning grafts (B1, B2) are shown from expt #20 (bank MCB4, Table S2), with matching left and right EFP grafts from one mouse. Hematoxylin and eosin staining of a graft cross-section, a composite graft-wide image of glucagon (red), somatostatin (green) and insulin (blue) expression, and higher magnification of the boxed region(s) are shown for each graft (in upper and lower order). The fasting serum C-peptide levels, 30 min and 60 min GSIS stimulation for this mouse at week 15 are indicated. Scale bars (H&E image): 3 mm.
(TIF)

Figure S12 **Histological and immunofluorescence analysis of CyT49-derived neo-pancreatic grafts.** Two representative functioning grafts (C1, C2) are shown from expt #20 (bank MCB4, Table S2), with matching left and right EFP grafts from one mouse. Hematoxylin and eosin staining of a graft cross-section, a composite graft-wide image of glucagon (red), somatostatin (green) and insulin (blue) expression, and higher magnification of the boxed region(s) are shown for each graft (in upper and lower order). The fasting serum C-peptide levels, 30 min and 60 min GSIS stimulation for this mouse at week 15 are indicated. Scale bars (H&E image): 3 mm.
(TIF)

Figure S13 **Histological and immunofluorescence analysis of CyT49-derived neo-pancreatic grafts.** Two representative functioning grafts (D1, D2) are shown from expt #20 (bank MCB4, Table S2), with matching left and right EFP grafts from one mouse. Hematoxylin and eosin staining of a graft cross-section, a composite graft-wide image of glucagon (red), somatostatin (green) and insulin (blue) expression, and higher magnification of the boxed region(s) are shown for each graft (D2 in upper and lower order). The fasting serum C-peptide levels, 30 min and 60 min GSIS stimulation for this mouse at week 15 are indicated. Scale bars (H&E image): 3 mm.
(TIF)

Figure S14 **Histological and immunofluorescence analysis of CyT49-derived neo-pancreatic grafts.** Two representative functioning grafts (E1, E2) are shown from expt #20 (bank MCB4, Table S2), with matching left and right EFP grafts from one mouse. Hematoxylin and eosin staining of a graft cross-section, a composite graft-wide image of glucagon (red), somatostatin (green) and insulin (blue) expression, and higher magnification of the boxed region(s) are shown for each graft (in upper and lower order). The fasting serum C-peptide levels, 30 min and 60 min GSIS stimulation for this mouse at week 15 are indicated. Scale bars (H&E image): 3 mm.
(TIF)

Table S1 Thaw and scaled expansion from CyT49 single cell banks.
(PDF)

Table S2 Scaled pancreatic differentiation runs.
(PDF)

Table S3 ELISA samples per *in vivo* GSIS plot in Figure S8.
(PDF)

Methods S1 Supplemental methods.
(DOC)
