# Supplementary figures and images for: A Scalable System for Production of Functional Pancreatic Progenitors from Human Embryonic Stem Cells
Source: PLoS One. 2012 May 18;7(5):e37004. doi: 10.1371/journal.pone.0037004 (PMC3356395; doi:10.1371/journal.pone.0037004)

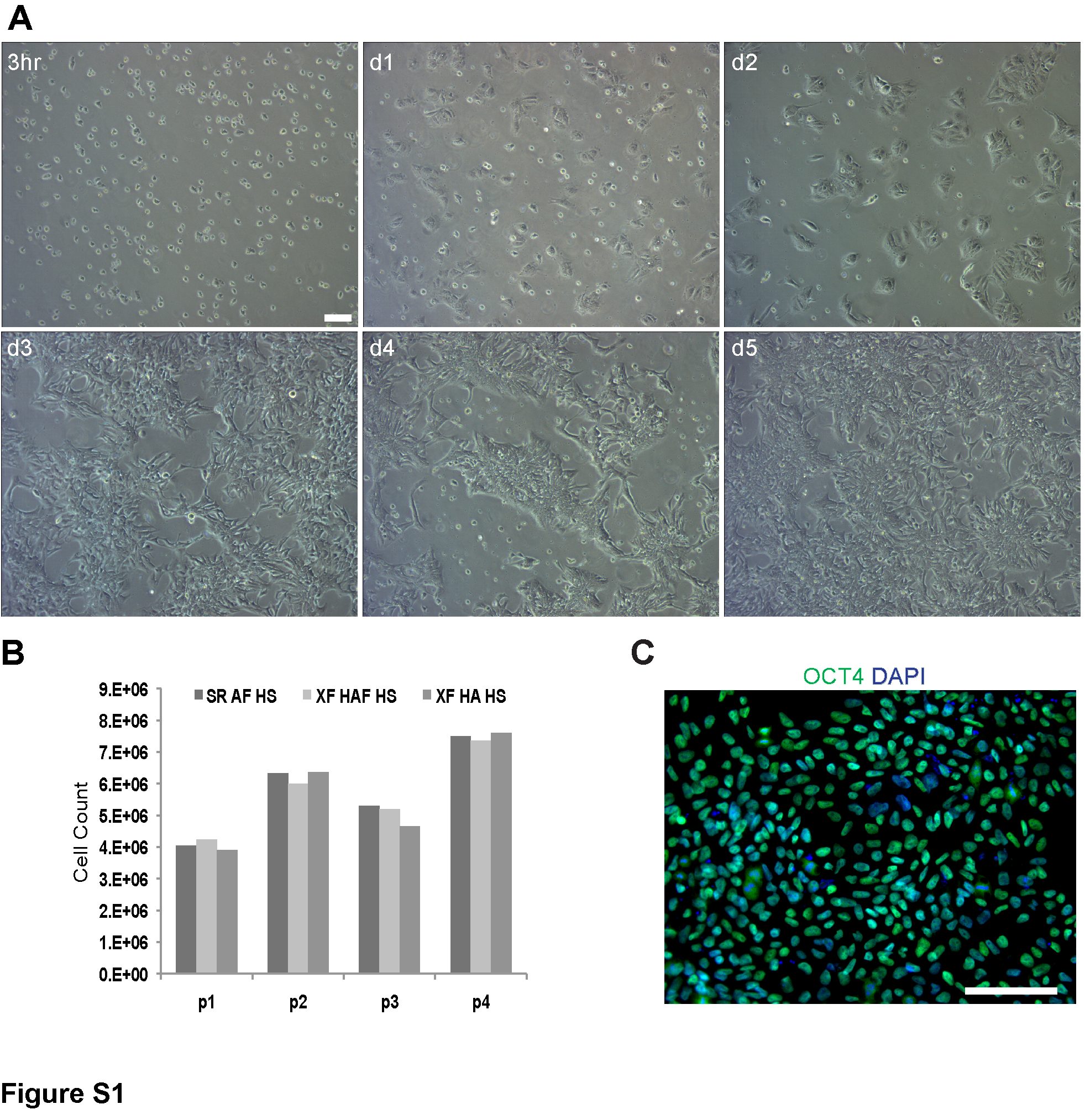

Supplement: Figure S1 — Scalable feeder-free culture system for CyT49 expansion. (A) Phase contrast images of single-cell passaged CyT49 at six time points after plating. Single plated cells (3 hr) migrate to form micro-colonies (d1), which proliferate to form a near confluent monolayer of hESC (d5). (B) Cell counts of serially passaged CyT49 cells in xeno-free culture media confirmed that addition of FGF2 was not required for long-term self-renewal under these conditions (XF HA vs XF HAF). A control condition (SR AF HS), which also supports self-renewal of undifferentiated hESC (data not shown) was included. The base media was DMEM/F12, and the cultures were passaged with Accutase and maintained in 6-well trays. XF: 10% xeno-free serum replacer, SR: 20% knockout serum replacer, H: 10 ng/mL heregulin-1β, A: 10 ng/mL Activin A, F: 10 ng/mL FGF2, HS: 10% soluble-phase human serum for attachment (first day of each passage only). (C) CyT49 maintained in XF HA for 10 passages retained uniform expression of OCT4. DAPI, 4′,6-diamidino-2-phenylindole. Scale bars: 100 µm. (TIF) [file pone.0037004.s001.tif]

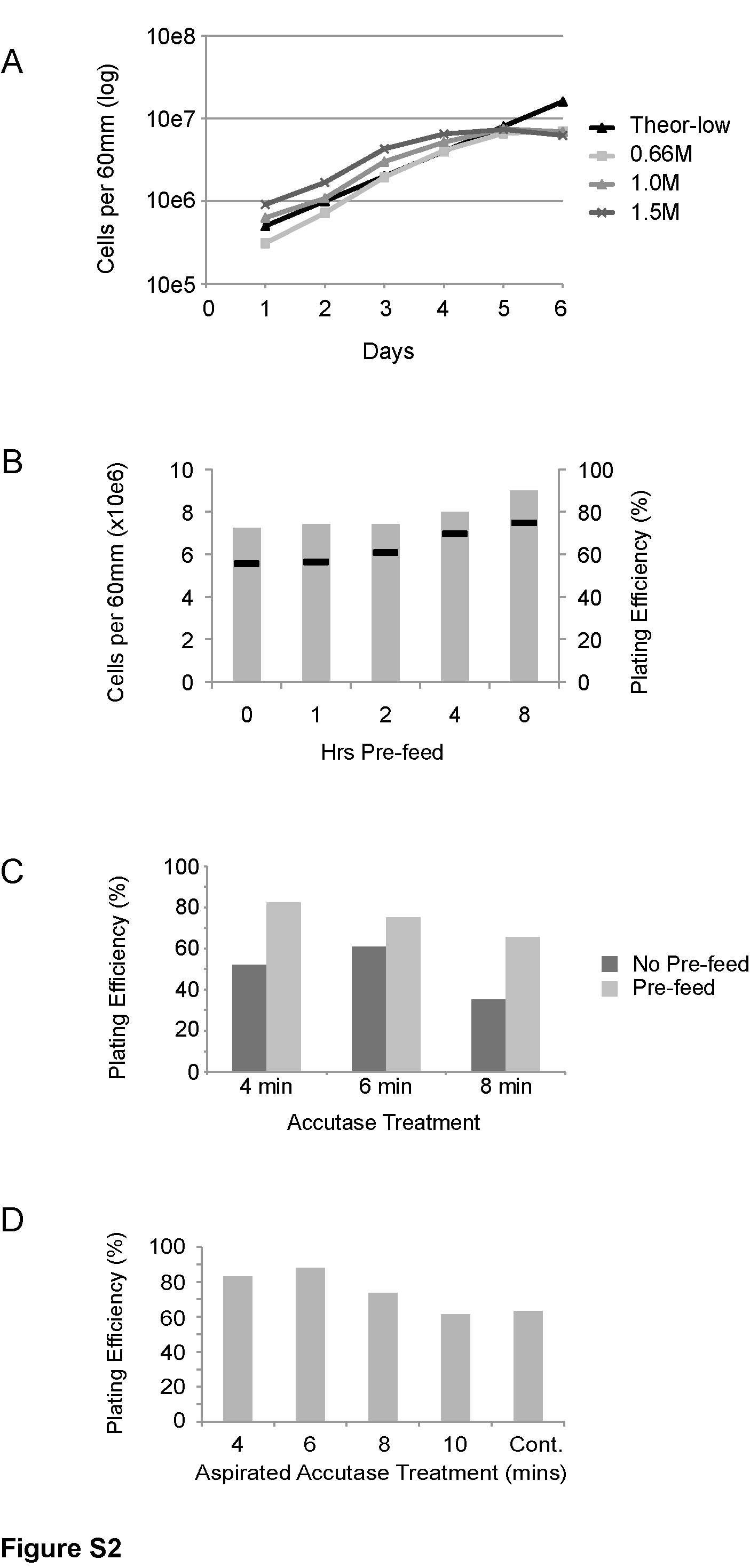

Supplement: Figure S2 — Optimization of the feeder-free culture system for scaled expansion of CyT49. (A) Determination of the cell yield 1–6 days after plating with three different densities (0.66, 1.0, or 1.5×10^6^ cells/60 mm dish, or 3.3, 5.0, or 7.6×10^4^ cells/cm2, respectively) demonstrated that near-exponential expansion occurred for the first four days. In comparison to a plot of theoretical-low expansion (plating 0.5×10^6^ cells/60 mm dish, 24 hr population doubling), the cell yield did not continue to increase after day four. Slowing of expansion was detected earliest, and was most prominent at, the highest plating density, suggesting that culture confluence may restrict further yield increases. These studies contributed to electing to use densities of 5×10^4^ cells/cm2 for a 3-day culture interval, and 3.3×10^4^ cells/cm2 for a 4-day culture interval during large-scale processes. (B) Pre-feeding of cultures prior to passaging improves plating efficiency. Cultures were fed with fresh media for 1, 2, 4, or 8 hrs prior to passaging (n = 2 dishes per time point). A moderate increase in cell yield was observed with pre-feeding (grey columns, left axis), as well as in plating efficiency (black bars, right axis). Plating efficiencies were determined by counting cultures 24 hrs post-plating. (C) The duration of Accutase dissociation was standardized to a 6-minute exposure by determining that it supported effective plating efficiencies for both pre-fed and non pre-fed cultures compared to a 4-, or 8-minute treatment. (D) In order to reduce the number of handling steps in scaled-expansion processes, we demonstrated that Accutase could still disaggregate cultures effectively if it was added, then immediately aspirated from vessels. The residual activity (“aspirated accutase”) was sufficient to disaggregate cultures and provide a plating efficiency of >85%. The plating efficiency of cultures grown in 60 mm dishes, pre-fed for 7 hrs, and then exposed to aspirated Accutase for 4, 6, 8, or 10 minutes a [file pone.0037004.s002.tif]

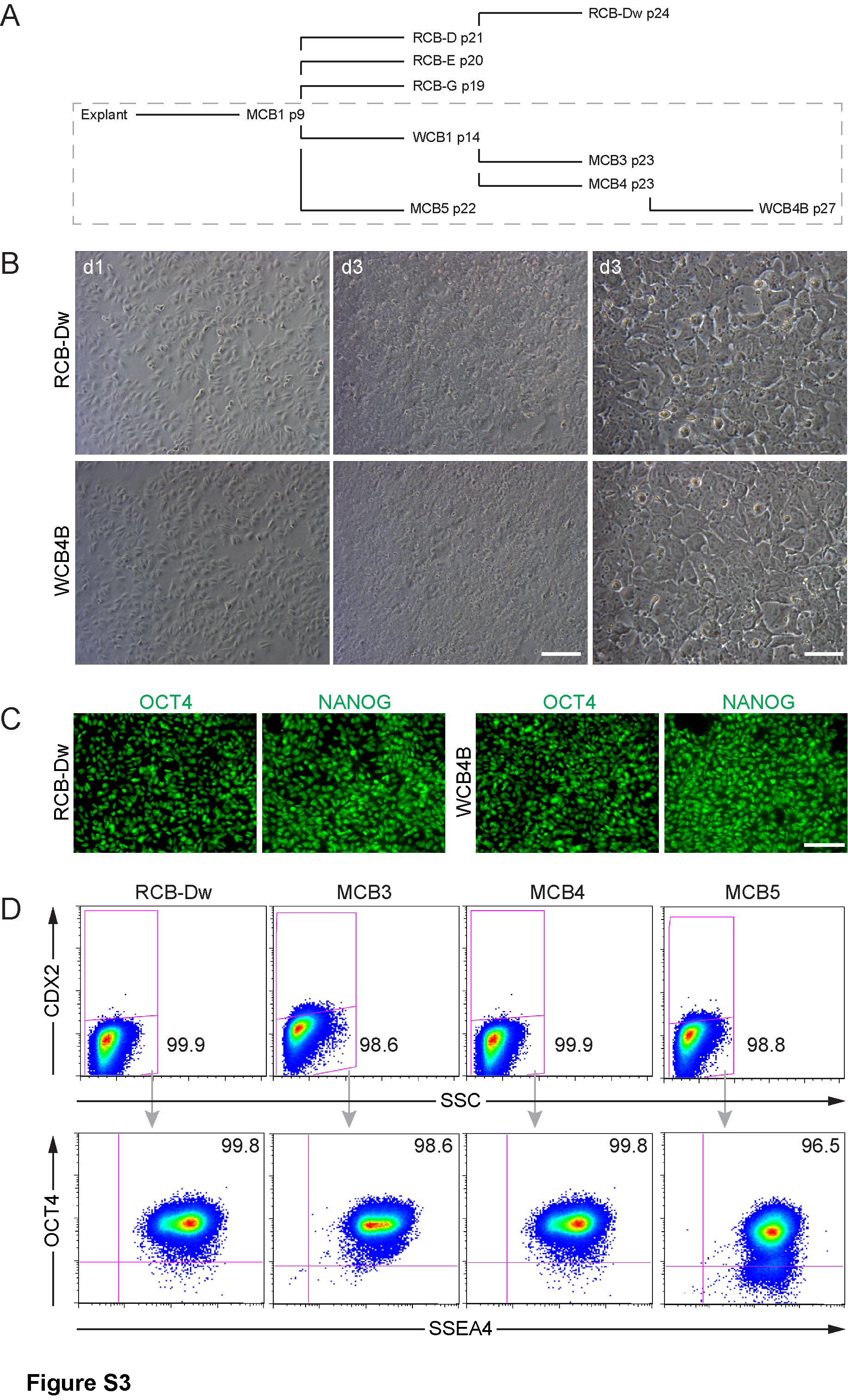

Supplement: Figure S3 — CyT49 cell banks. (A) Derivation and genealogy of CyT49 cell banks. Stepped lines indicate a thaw and expansion from the indicated bank. cGMP conditions indicated by the dashed box. (B) Phase contrast images of thawed CyT49 cultures from the RCB-Dw and WCB4B large-scale cell banks, imaged with a 20× objective at day 1 or 3 (left, center panels. Scale bar: 200 µm), or with a 40× objective at day 3 (right panels. Scale bar: 50 µm). Thawed cultures exhibited a primarily undifferentiated morphology. (C) Immunofluorescence analysis of cultures one passage after thaw, demonstrating maintenance of expression of OCT4 and NANOG. Scale bar: 100 µm. (D) Flow cytometric analysis of thawed cultures from RCB-Dw, MCB3, MCB4 and MCB5, co-stained with anti-CDX2, anti-OCT4, and anti-SSEA4. The analyses were first gated on the CDX2dim population (upper), followed by plotting OCT4/SSEA4 co-positive cells (lower). The population-wide (total) percentage of gated cells is shown. Typically, >98% of the population was comprised of CDX2dim/OCT4+/SSEA4+ undifferentiated cells. SSC: side scatter. Methods for labeling and cytometry were essentially as described previously [7]. (TIF) [file pone.0037004.s003.tif]

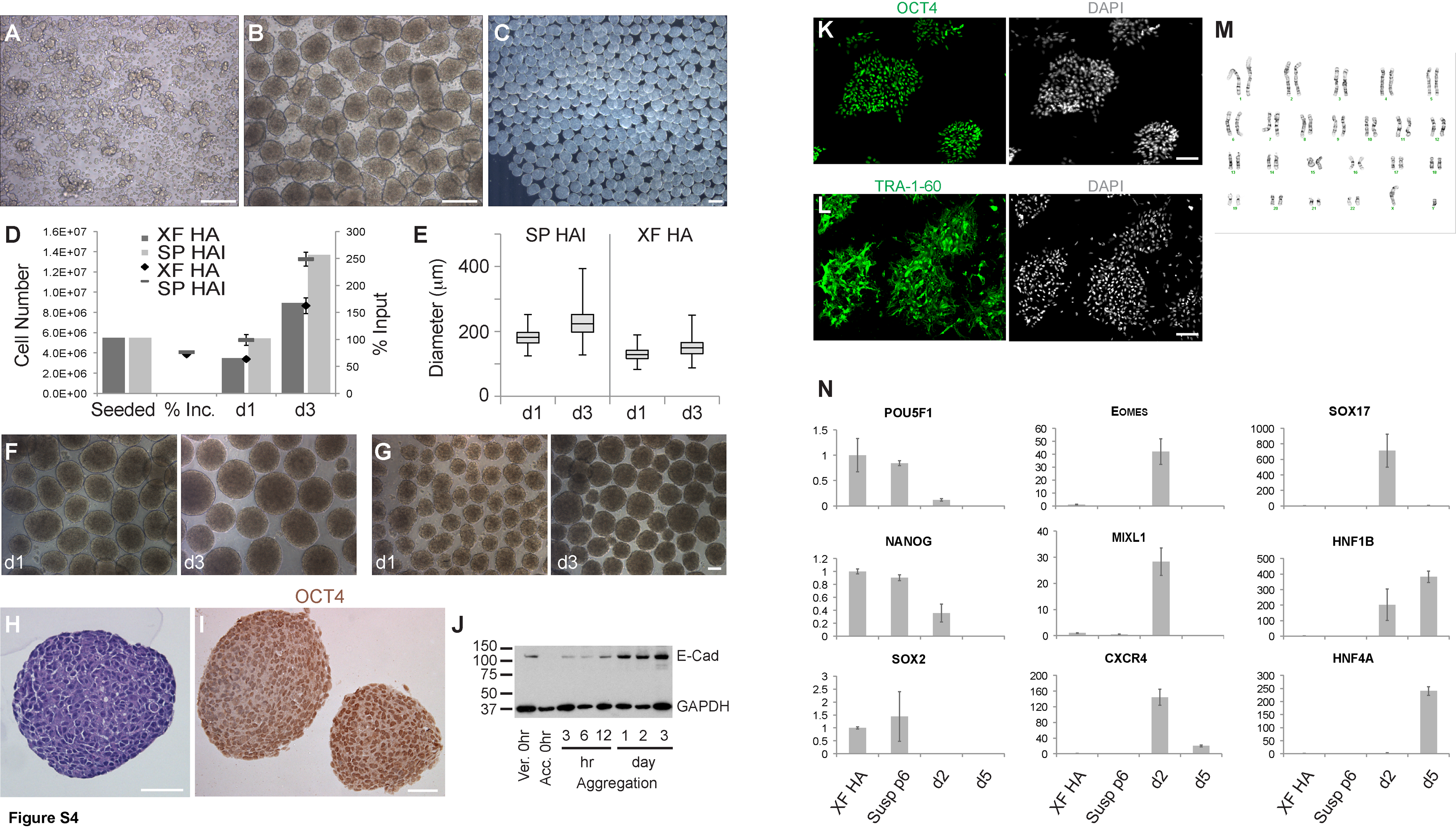

Supplement: Figure S4 — Aggregation and serial culture of hESC in suspension as undifferentiated aggregates. (A) Single cell suspension of CyT49 after 4 hrs of aggregation with rotational culture showing initial cluster formation, and (B) aggregates after 24 hrs in XF HA media. The BG02 hESC line was also used in some of these studies [47]. (C) BG02 aggregates after 2 days culture in StemPro medium. (D) Incorporation efficiency and expansion of CyT49 in aggregates in XF HA or SP HAI medium (left axis: columns = cell counts; right axis: diamond and grey bar = % of input cells). Cells were aggregated in triplicate, in 6-well trays, in 5.5 mL at 106 cells/mL (seeded). The incorporation efficiencies were estimated after 24 hrs by two methods. First, counting the number of live unincorporated cells and extrapolating, indicated that 72±3.2% and 76±1.5% of input cells were incorporated (% Inc.) for XF HA and SP HAI conditions, respectively (mean ± SD, n = 3). Secondly, cell counts of dissociated aggregates (d1) indicated that they contained 63.5±2.2% and 98.9±10.1% of input cells for XF HA and SP HAI conditions, respectively. After 3 days of expansion (d3) aggregates contained 162.4±14.7% and 248.7±12.7% of input cells for XF HA and SP HAI conditions, respectively. (E) Diameters of undifferentiated CyT49 aggregates. Aggregates were imaged under 3× magnification on a dissecting microscope and diameters were measured with the ImagePro software. Triplicate wells were imaged and 100 aggregates were measured for each image (n = 3×100). The box plots show the median, second and third quartile (box), max and min values for each condition. (F) Representative SP HAI aggregates from (D,E), and (G) XF HA aggregates from (D,E), imaged with an inverted microscope. (H) Hematoxylin and eosin and (I) anti-OCT4 stained sections of BG02 aggregates grown in StemPro medium demonstrating undifferentiated morphology, lack of overt differentiation, layer formation or cavitation, and uniform expression of OCT4. (J) West [file pone.0037004.s004.tif]

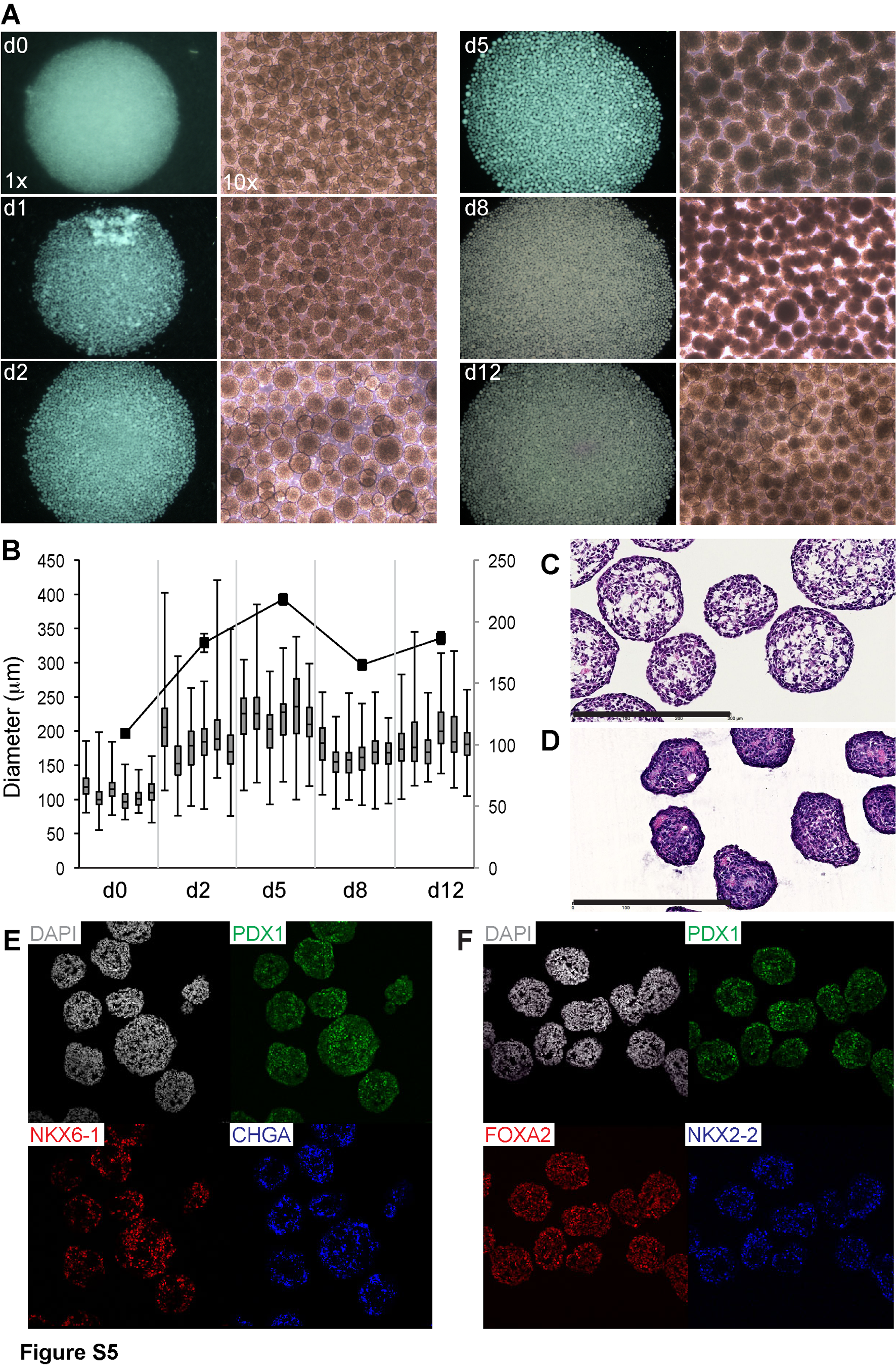

Supplement: Figure S5 — Characterization of pancreatic differentiation in rotational suspension culture. (A) Low magnification (dissecting microsope 1× objective) and phase contrast (10× objective) imaging of undifferentiated aggregates (d0) and differentiating aggregates at d1, 2, 5, 8 and 12. Some large-scale differentiations, including this example, were pooled at the start of Stage-3, consolidating to half as many total wells. (B) Box plots of diameter measurements of six independent scaled differentiation runs (left to right: Table S2, Expt #4, 9, 10, 21, 27, 32) show the median, second and third quartile (box), max and min values for each data set (left axis). The mean ± SEM for the full data is shown (black squares) on the right axis (µm), with the scale shifted for clarity. (C) Additional examples of H&E staining of sectioned aggregates at d5 and (D) d12 of differentiation. Scale bars: 300 µm. (E,F) Additional examples of immunofluorescence analysis of sectioned d12 differentiated aggregates. (E) Expression of PDX1, NKX6-1 and CHGA; (F) expression of PDX1, FOXA2 and NKX2-2. (TIF) [file pone.0037004.s005.tif]

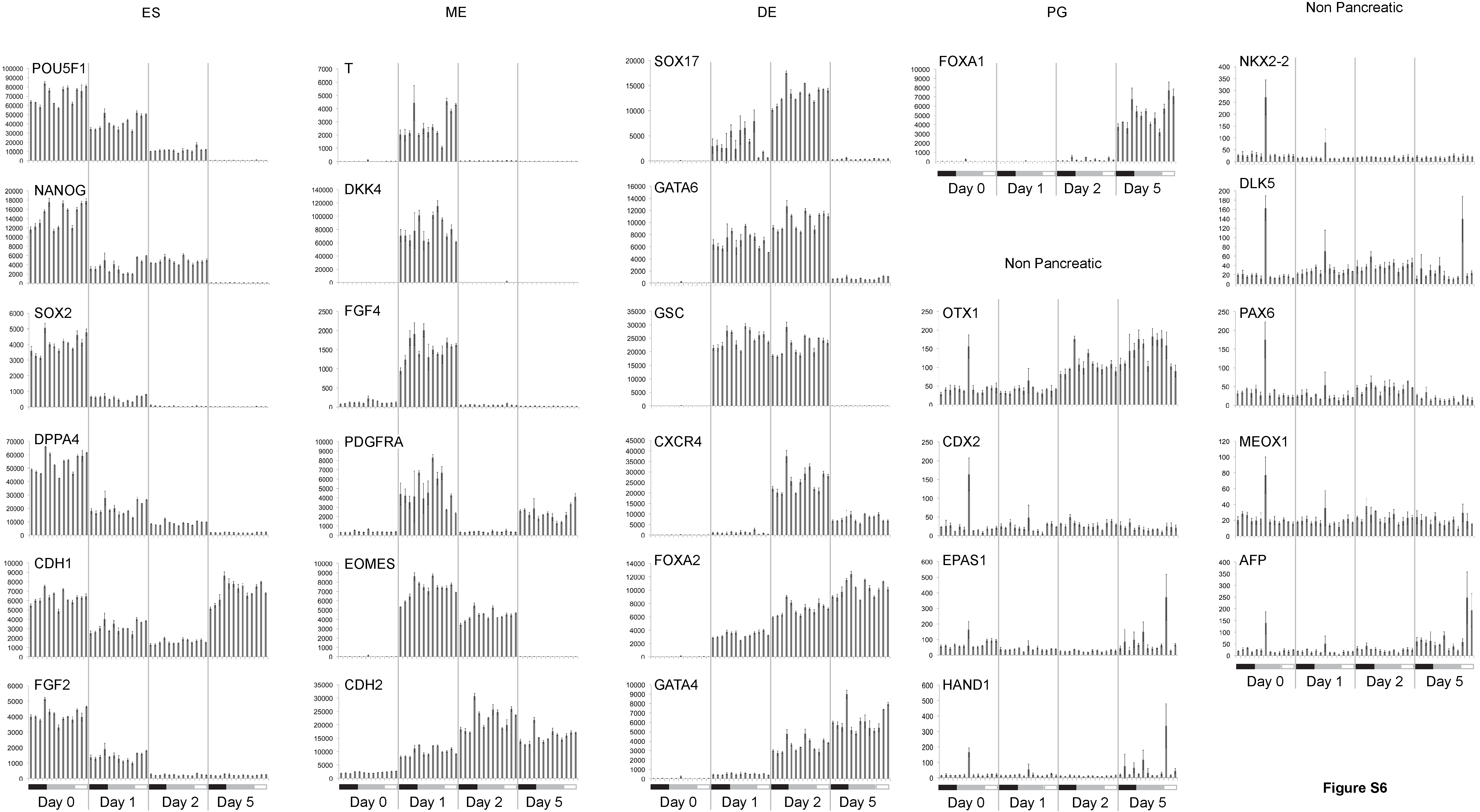

Supplement: Figure S6 — Digital mRNA profiling of Stages-1 and -2 of scaled pancreatic differentiation runs. Markers are displayed as groups depicting undifferentiated cells (ES), mesendoderm (ME), definitive endoderm (DE), primitive gut tube (PG), and non-pancreatic off target probes for the undifferentiated aggregates (d0) and the early stages of differentiation (d1, 2, 5). The plots of the C13 group are ordered according to CyT49 cell bank (left to right): black bar (MCB4: Expt #18–21), grey bar (RCB–Dw: Expt #25–30), open grey bar (WCB4B: Expt #35–37). The average and standard deviation of three biological replicates are plotted. (TIF) [file pone.0037004.s006.tif]

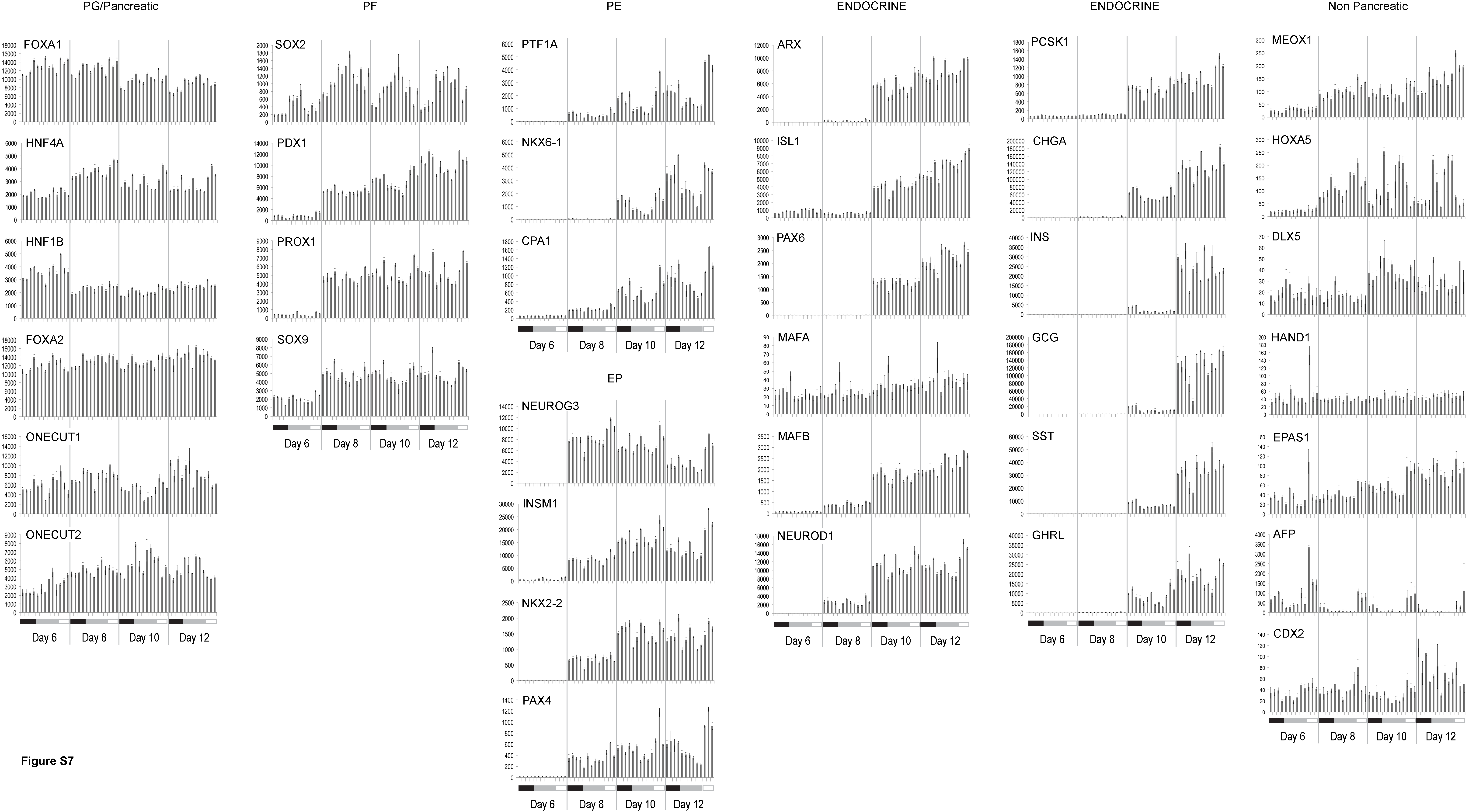

Supplement: Figure S7 — Digital mRNA profiling of Stages-3 and -4 of scaled pancreatic differentiation runs. Markers are displayed as groups depicting primitive gut/pancreatic (PG/pancreatic), posterior foregut (PF), pancreatic endoderm (PE), endocrine progenitor (EP), endocrine, and non-pancreatic off target probes for the later stages of differentiation (d6, 8, 10, 12). The plots of the C13 group are ordered according to CyT49 cell bank (left to right): black bar (MCB4: Expt #18–21), grey bar (RCB-Dw: Expt #25–30), open grey bar (WCB4B: Expt #35–37). The average and standard deviation of three biological replicates are plotted. (TIF) [file pone.0037004.s007.tif]

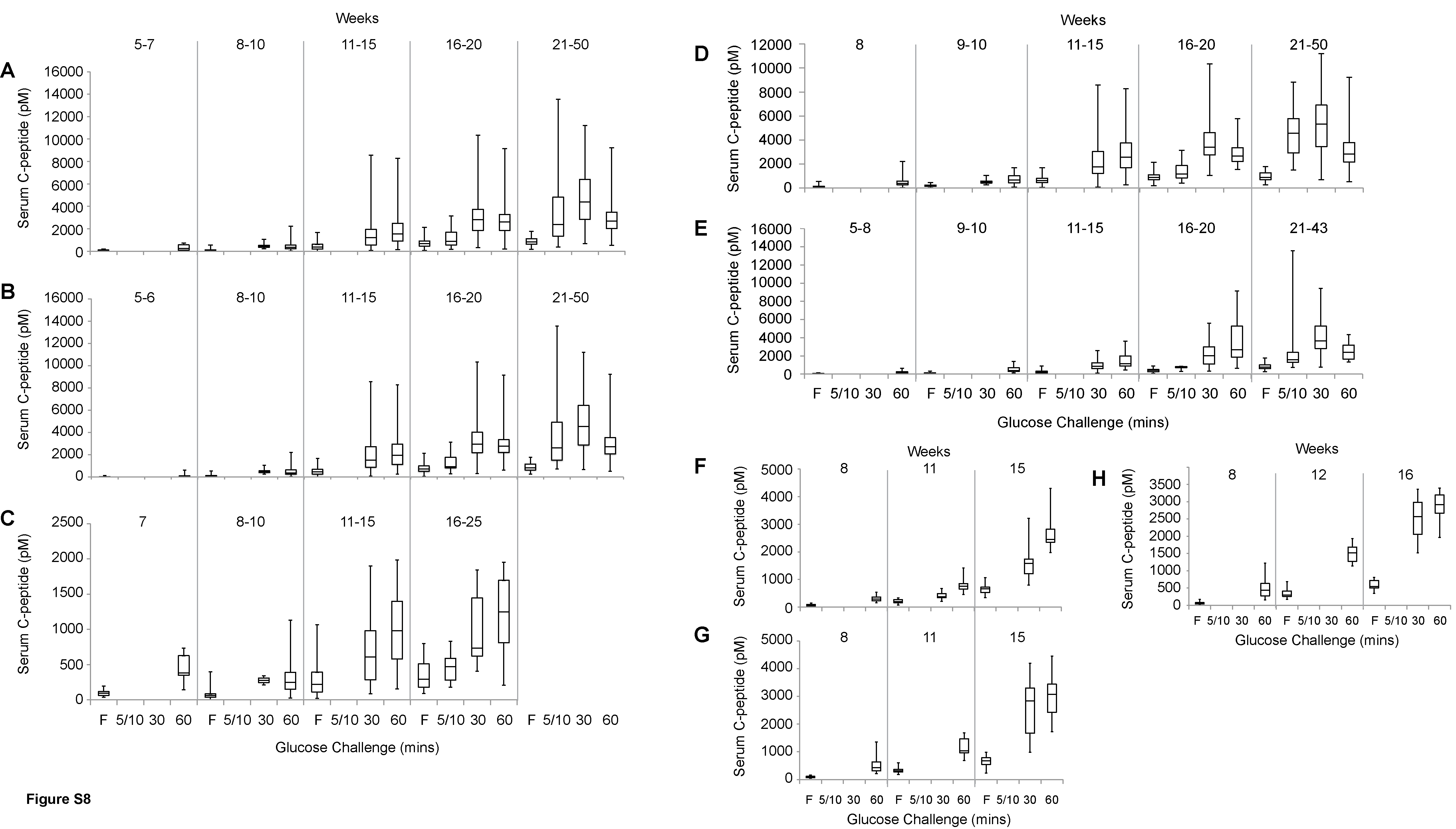

Supplement: Figure S8 — In vivo function of engrafted pancreatic differentiations. GSIS response in (A) all functioning engrafted animals (n = 228), (B) high functioning engrafted animals (n = 166 mice), (C) the “partially protected” group (n = 62), and the high functioning animals split into their respective starting CyT49 banks: (D) RCB-D (n = 75), (E) RCB-Dw (n = 37), (F) MCB3 (n = 17), (G) MCB4 (n = 23) and (H) MCB5 (n = 15). The data are presented as selected weeks post-engraftment. Each group shows fasting (F), 5 or 10 minute (5/10), 30 minute, and 60 minute serum human C-peptide measurements. The box plots show the median, second and third quartile (box), max and min values for serum human C-peptide (pM). Empty plots indicate that no data were collected. The number of ELISA samples for each data set is indicated in Table S3. (TIF) [file pone.0037004.s008.tif]

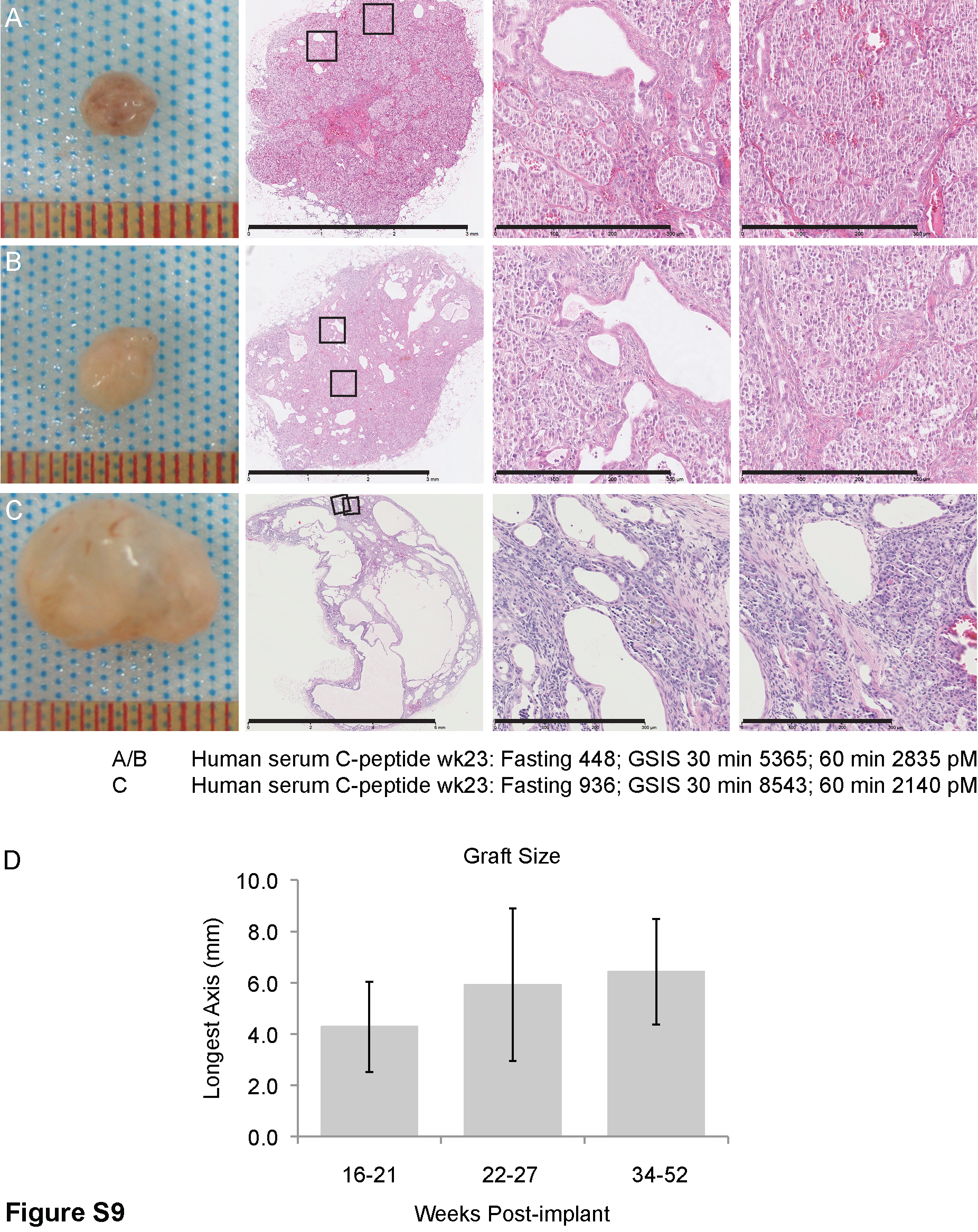

Supplement: Figure S9 — Histological and size analysis of CyT49-derived neo-pancreatic grafts. Images of explanted grafts and matching H&E stained sections: (A) left and (B) right grafts from one mouse, 25 weeks post-transplant, derived from expt #18 (MCB3). (C) 26 weeks post-transplant graft from expt #3 (RCB-D). The boxed regions are magnified in the adjacent columns (in left and right order). The week-23 fasting serum C-peptide levels, 30 min and 60 min GSIS stimulation for these representative mice are indicated. Scale in left column indicated by mm ruler. Scale bars for H&E panels: left column (A,B) 3 mm, (C) 6 mm; right two columns 300 µm. (D) A total of 191 explanted grafts from 112 mice (145 grafts/84 mice from the high functioning group; 46 grafts/28 mice from the partially protected group) were measured prior to histological processing and were grouped according to weeks post-implantation: Weeks 16–21 (n = 51 mice, 95 grafts); Weeks 22–27 (n = 45 mice, 75 grafts); Weeks 34–52 (n = 16 mice, 21 grafts). The average and standard deviation of the longest axis was plotted. Total: 5.2 mm±2.5 mm; max = 15.0 mm; min = 2.0 mm. (TIF) [file pone.0037004.s009.tif]

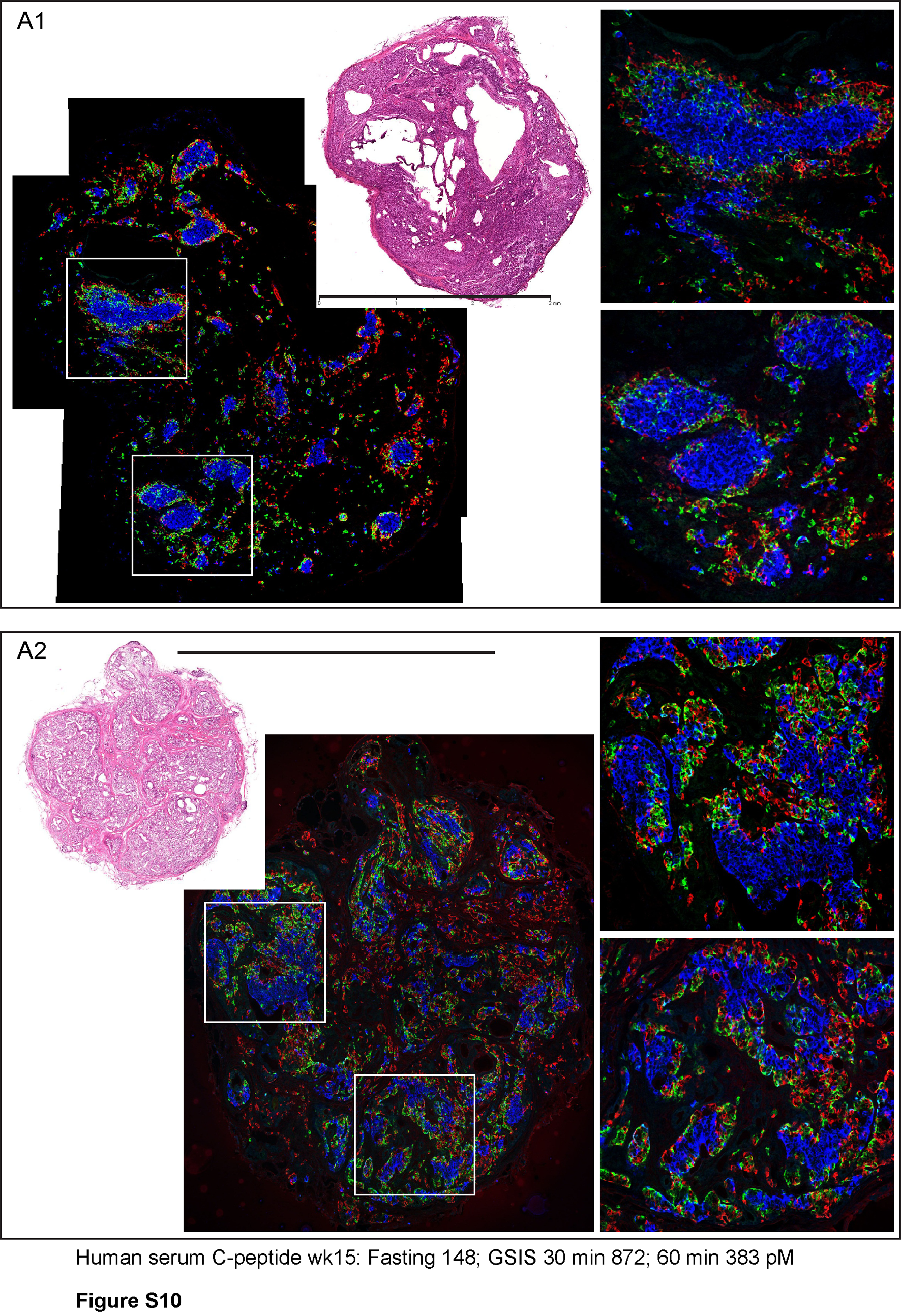

Supplement: Figure S10 — Histological and immunofluorescence analysis of CyT49-derived neo-pancreatic grafts. Two representative functioning grafts (A1, A2) are shown from expt #20 (bank MCB4, Table S2), with matching left and right EFP grafts from one mouse. Hematoxylin and eosin staining of a graft cross-section, a composite graft-wide image of glucagon (red), somatostatin (green) and insulin (blue) expression, and higher magnification of the boxed region(s) are shown for each graft (in upper and lower order). The fasting serum C-peptide levels, 30 min and 60 min GSIS stimulation for this mouse at week 15 are indicated. Scale bars (H&E image): 3 mm. (TIF) [file pone.0037004.s010.tif]

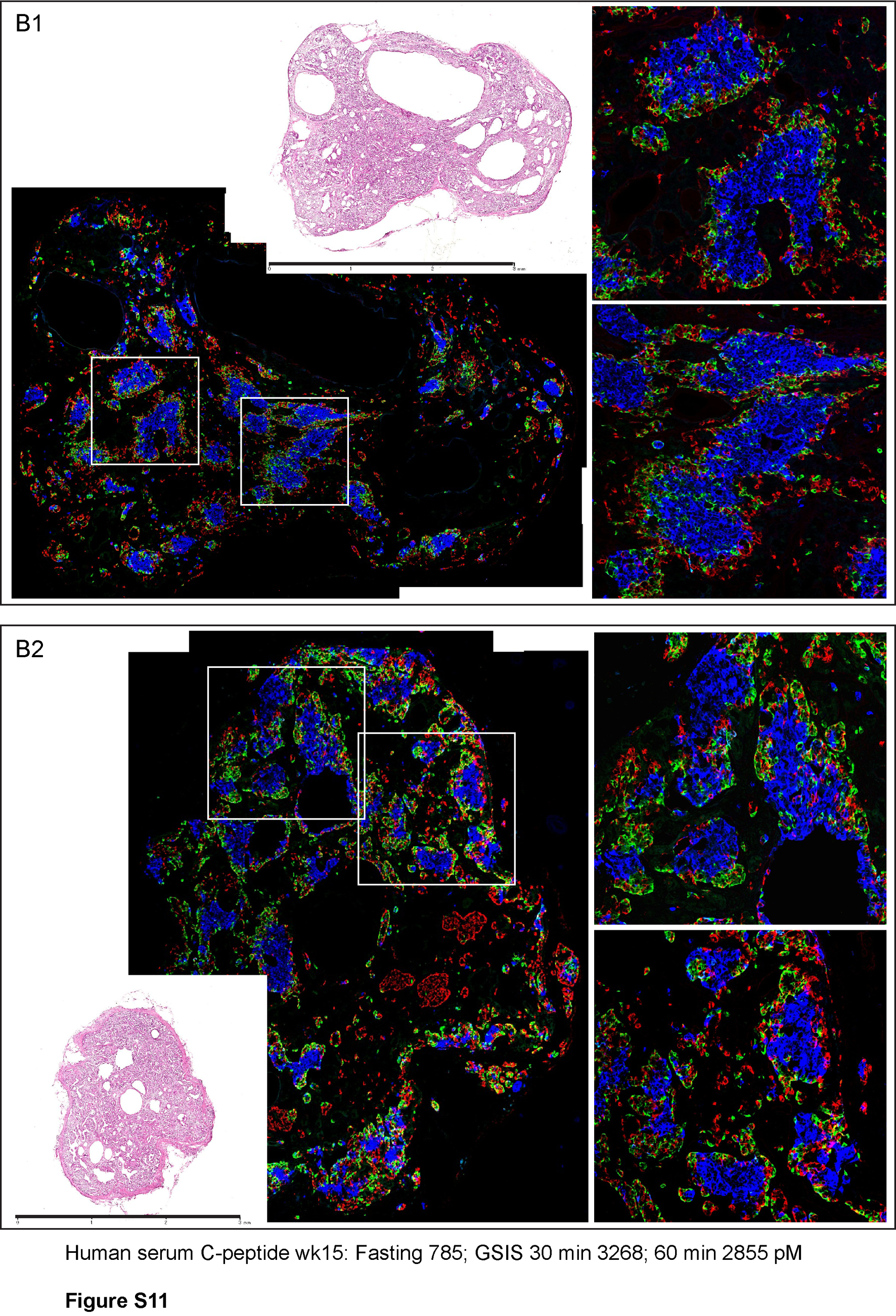

Supplement: Figure S11 — Histological and immunofluorescence analysis of CyT49-derived neo-pancreatic grafts. Two representative functioning grafts (B1, B2) are shown from expt #20 (bank MCB4, Table S2), with matching left and right EFP grafts from one mouse. Hematoxylin and eosin staining of a graft cross-section, a composite graft-wide image of glucagon (red), somatostatin (green) and insulin (blue) expression, and higher magnification of the boxed region(s) are shown for each graft (in upper and lower order). The fasting serum C-peptide levels, 30 min and 60 min GSIS stimulation for this mouse at week 15 are indicated. Scale bars (H&E image): 3 mm. (TIF) [file pone.0037004.s011.tif]

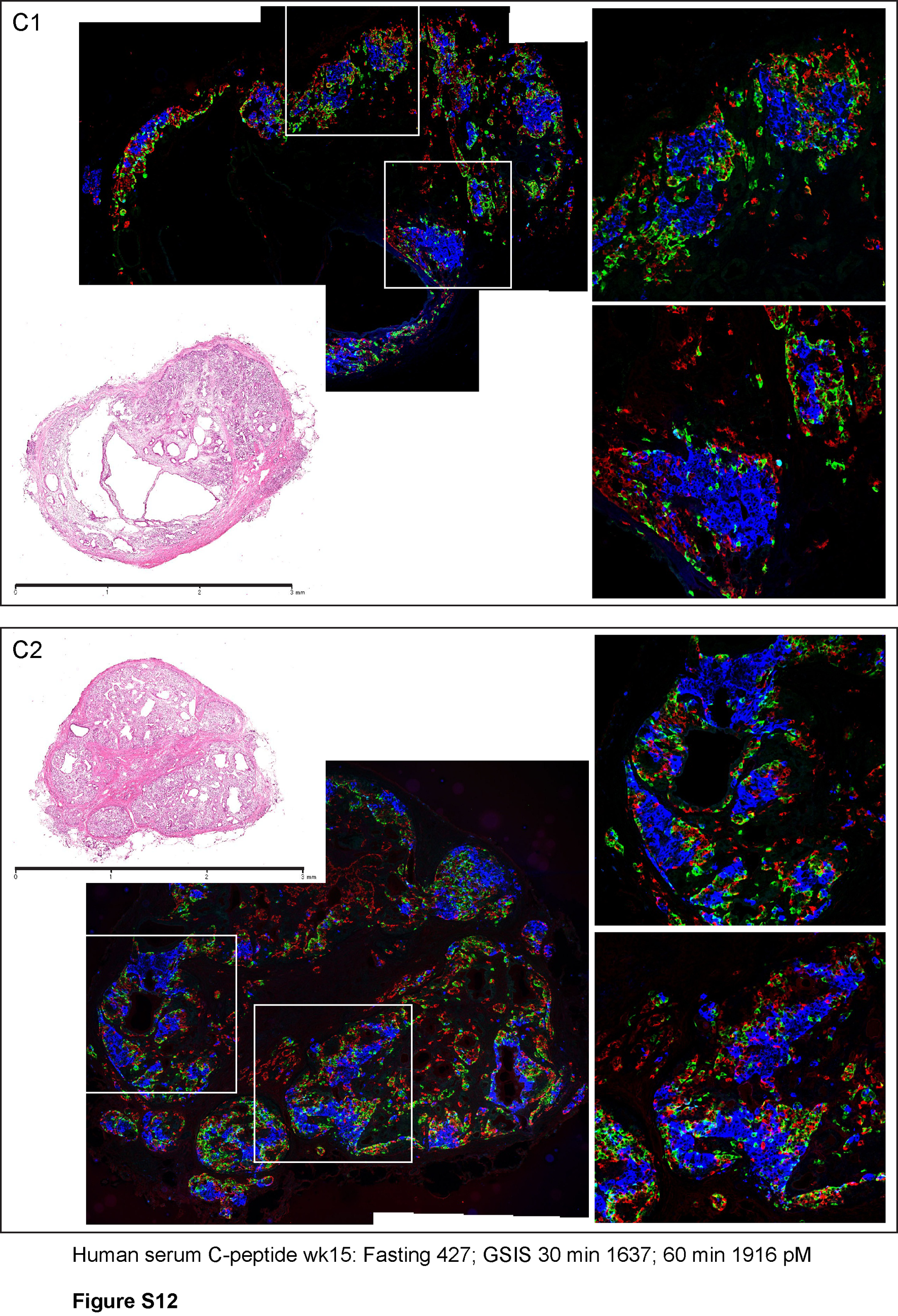

Supplement: Figure S12 — Histological and immunofluorescence analysis of CyT49-derived neo-pancreatic grafts. Two representative functioning grafts (C1, C2) are shown from expt #20 (bank MCB4, Table S2), with matching left and right EFP grafts from one mouse. Hematoxylin and eosin staining of a graft cross-section, a composite graft-wide image of glucagon (red), somatostatin (green) and insulin (blue) expression, and higher magnification of the boxed region(s) are shown for each graft (in upper and lower order). The fasting serum C-peptide levels, 30 min and 60 min GSIS stimulation for this mouse at week 15 are indicated. Scale bars (H&E image): 3 mm. (TIF) [file pone.0037004.s012.tif]

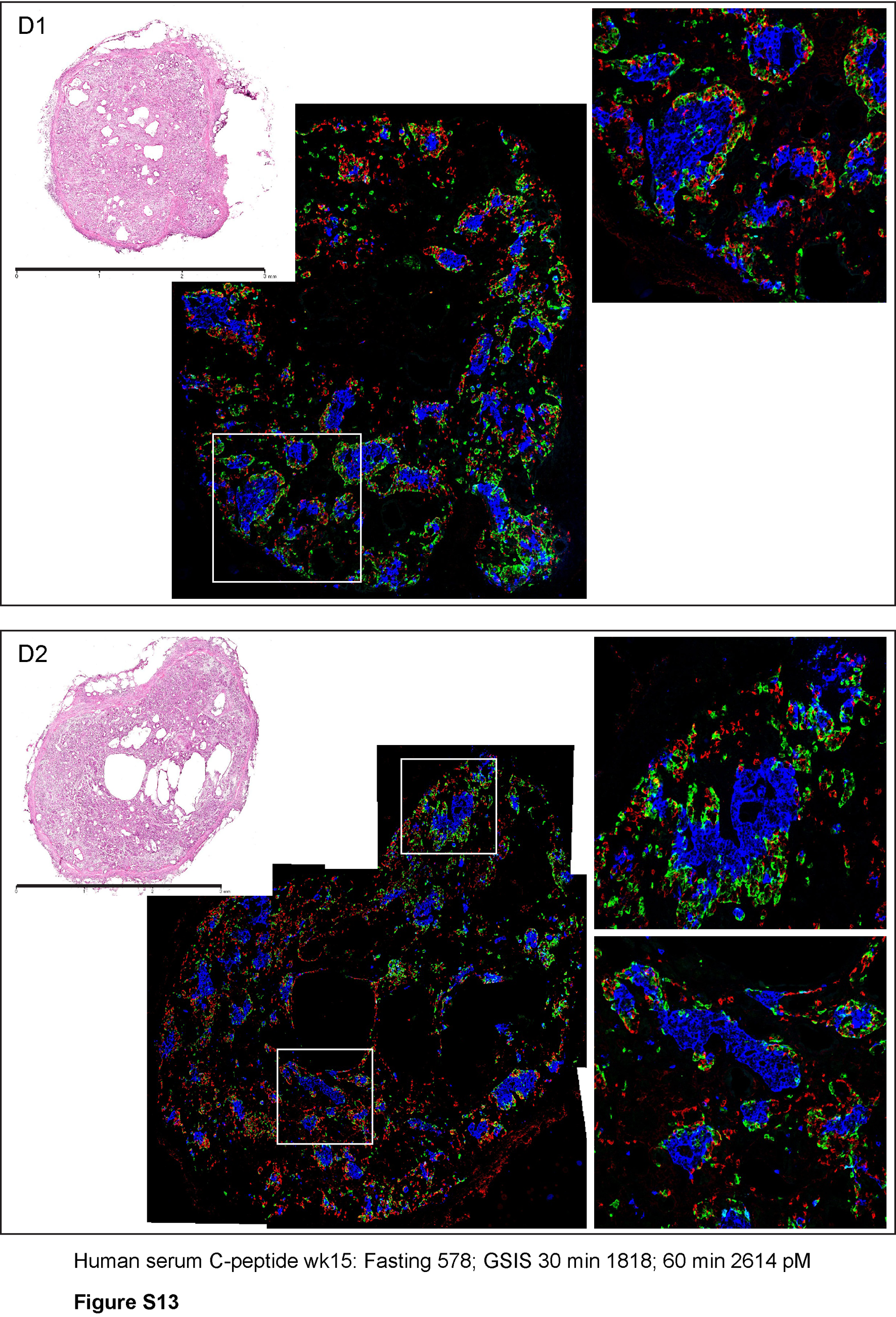

Supplement: Figure S13 — Histological and immunofluorescence analysis of CyT49-derived neo-pancreatic grafts. Two representative functioning grafts (D1, D2) are shown from expt #20 (bank MCB4, Table S2), with matching left and right EFP grafts from one mouse. Hematoxylin and eosin staining of a graft cross-section, a composite graft-wide image of glucagon (red), somatostatin (green) and insulin (blue) expression, and higher magnification of the boxed region(s) are shown for each graft (D2 in upper and lower order). The fasting serum C-peptide levels, 30 min and 60 min GSIS stimulation for this mouse at week 15 are indicated. Scale bars (H&E image): 3 mm. (TIF) [file pone.0037004.s013.tif]

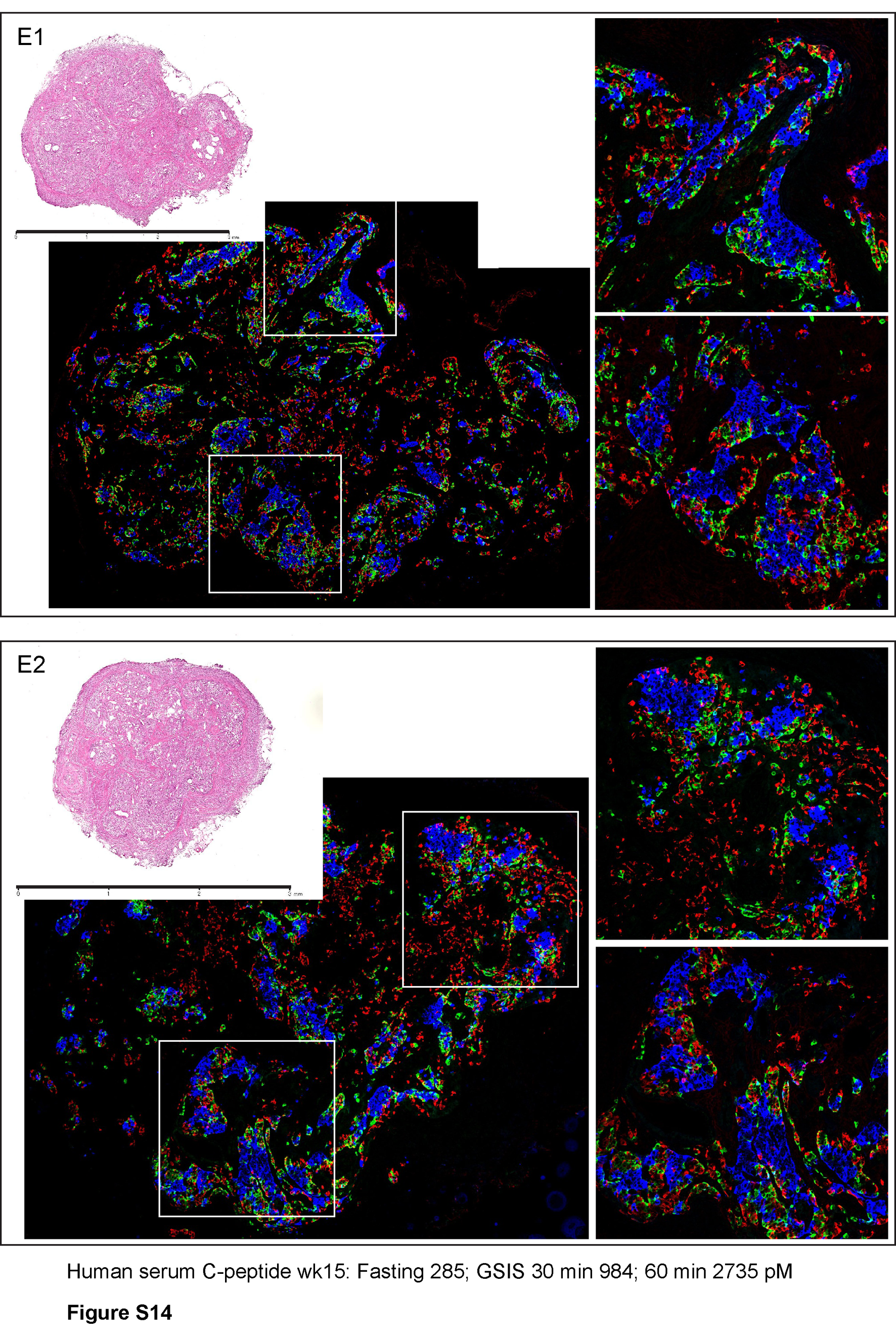

Supplement: Figure S14 — Histological and immunofluorescence analysis of CyT49-derived neo-pancreatic grafts. Two representative functioning grafts (E1, E2) are shown from expt #20 (bank MCB4, Table S2), with matching left and right EFP grafts from one mouse. Hematoxylin and eosin staining of a graft cross-section, a composite graft-wide image of glucagon (red), somatostatin (green) and insulin (blue) expression, and higher magnification of the boxed region(s) are shown for each graft (in upper and lower order). The fasting serum C-peptide levels, 30 min and 60 min GSIS stimulation for this mouse at week 15 are indicated. Scale bars (H&E image): 3 mm. (TIF) [file pone.0037004.s014.tif]
