# Supplementary material for: A Scalable System for Production of Functional Pancreatic Progenitors from Human Embryonic Stem Cells
Source: PLoS One. 2012 May 18;7(5):e37004. doi: 10.1371/journal.pone.0037004 (PMC3356395; doi:10.1371/journal.pone.0037004)
Supplement: Table S1 — Thaw and scaled expansion from CyT49 single cell banks. (PDF) [file pone.0037004.s015.pdf]

Table S1

Bank	αExpt.	αAnalysis	Passage	Vessel Type	No. of Vessels	Total γS.A.	Cells/cm^2	Total No. of Cells	δViability (%)
MCB4	21	35	thaw (p23)	vial	---	---	---	$9.3x10^6$	86.5
			p24	T75	2	150	245,333	$3.7x10^7$	97.8
			p25	T175	2	350	379,143	$1.3x10^8$	98.6
			p26	T175	8	1400	367,500	$5.1x10^8$	98.1
			p27	5 Stack	3	9540	279,245	$2.7x10^9$	96.9
WCB4B	35	53	thaw (p27)	vial	---	---	---	$7.3x10^6$	90.4
			p28	T75	1	75	295,467	$2.2x10^7$	98.6
			p29	T175	2	350	388,571	$1.4x10^8$	97.6
			p30	T175	10	1750	305,143	$5.3x10^8$	97.8
			p31	5 Stack	1	3180	232,704	$7.4x10^8$	98.1
	36	54	thaw (p27)	vial	---	---	---	$7.3x10^6$	92.4
			p28	T75	1	75	291,200	$2.2x10^7$	99.1
			p29	T175	1	175	402,286	$7.0x10^7$	97.8
			p30	T175	2	350	370,286	$1.3x10^8$	98.3
			p31	5 Stack	1	3180	220,755	$7.0x10^8$	97.7
	37	55	thaw (p27)	vial	---	---	---	$9.1x10^6$	96.3
			p28	T75	1	75	236,800	$1.8x10^7$	98.4
			p29	T175	1	175	392,229	$6.9x10^7$	97.2
			p30	T175	2	350	400,286	$1.4x10^8$	98.8
			p31	5 Stack	1	3180	238,994	$7.6x10^8$	97.8

αListed in Table S2
γS.A., surface area (cm^2)
δLive/live+dead count at harvest
