# Supplementary material for: A Scalable System for Production of Functional Pancreatic Progenitors from Human Embryonic Stem Cells
Source: PLoS One. 2012 May 18;7(5):e37004. doi: 10.1371/journal.pone.0037004 (PMC3356395; doi:10.1371/journal.pone.0037004)
Supplement: Table S2 — Scaled pancreatic differentiation runs. (PDF) [file pone.0037004.s016.pdf]

Table S2

CHGA	neg	pos	neg	neg
NKX6-1	pos	+/-	neg	neg
PDX1	+/-	+/-	pos	neg

Bank	Expt.[γ κ]	Analysis[χ]	PE	Endocrine	PDX1-only	Residual	Karyotype[λ]
**RCB-D**	1	1	33.8	55.7	9.4	1.1	46,XY
		[δ]2	37.5	53.7	8.0	0.8	
	2	3	23.0	65.0	10.5	1.5	46,XY
	3	4	29.9	54.2	13.9	2.0	46,XY
	4	5	33.0	52.5	11.6	2.9	NR[α]
		[δ]6	35.6	46.7	15.4	2.3	
	5	7	22.0	60.2	16.1	1.7	
	6	[δ]8	30.7	42.0	22.0	5.3	46,XY
	7	9	27.6	42.3	25.5	4.6	46,XY
	8	10	22.6	64.5	11.1	1.8	46,XY
		11	25.9	60.9	10.3	2.9	
		12	30.8	59.1	8.0	2.1	
		13	27.9	59.2	10.4	2.5	
	9	14	23.4	61.8	12.4	2.4	46,XY[φ]
	10	15	26.8	53.2	18.0	2.0	46,XY
	11	16	33.5	42.0	22.2	2.3	46,XY
	12	17	19.6	59.5	17.0	3.9	46,XY
	13[ξ]	18	23.3	62.5	12.2	2.0	46,XY
	14	19	21.5	55.0	20.3	3.2	46,XY

**mean**	**27.8**	**55.3**	**14.4**	**2.5**
**median**	**27.6**	**55.7**	**12.4**	**2.3**
**min**	**19.6**	**42.0**	**8.0**	**0.8**
**max**	**37.5**	**65.0**	**25.5**	**5.3**
**n=19**				

Bank	Expt.	Analysis	PE	Endocrine	PDX1-only	Residual	Karyotype
**MCB3**	15	20	33.6	56.6	8.6	1.1	46,XY
		21	30.2	59.5	9.1	1.2	
	16	22	26.3	61.6	10.8	1.3	46,XY
		23	35.0	54.9	8.7	1.4	
	17	24	27.2	60.7	11.0	1.0	46,XY
		25	33.0	54.7	11.2	1.2	

**mean**	**30.9**	**58.0**	**9.9**	**1.2**
**median**	**31.6**	**58.1**	**10.0**	**1.2**
**min**	**26.3**	**54.7**	**8.6**	**1.0**
**max**	**35.0**	**61.6**	**11.2**	**1.4**
**n=6**				

Bank	Expt.	Analysis	PE	Endocrine	PDX1-only	Residual	Karyotype
**MCB4**	18[η]	26	33.6	50.6	15.4	0.3	46,XY
		27	32.1	50.1	16.8	1.0	
	19[η]	28	29.5	53.2	16.1	1.2	46,XY
		29	29.8	52.0	17.1	1.0	
	20[η]	30	34.6	50.9	14.0	0.6	46,XY
		31	36.5	46.4	16.2	0.9	
	21[ηξ]	32	40.7	44.5	14.2	0.6	46,XY

**mean**	**33.9**	**49.7**	**15.7**	**0.8**
**median**	**33.6**	**50.6**	**16.1**	**0.9**
**min**	**29.5**	**44.5**	**14.0**	**0.3**
**max**	**40.7**	**53.2**	**17.1**	**1.2**
**n=7**				

**MCB5**	22	33	35.3	47.7	16.0	1.0	46,XY[φ]
	23	34	33.6	48.7	16.1	1.6	46,XY
	24	35	32.6	50.5	15.9	1.0	46,XY
		**mean**	**33.8**	**49.0**	**16.0**	**1.2**	
		**median**	**33.6**	**48.7**	**16.0**	**1.0**	
		**min**	**32.6**	**47.7**	**15.9**	**1.0**	
		**max**	**35.3**	**50.5**	**16.1**	**1.6**	
		**n=3**					
**RCB-Dw**	25[η]	36	35.9	54.4	8.3	1.4	46,XY[φ]
		37	21.3	49.6	27.2	1.9	
	26[η]	38	22.2	58.1	17.3	2.4	46,XY
	27[η]	39	32.4	51.7	14.7	1.2	46,XY
	28[η]	[δ]40	41.2	51.3	6.6	1.0	46,XY
	29[η]	41	15.6	51.4	31.7	1.3	46,XY
	30[η]	42	37.7	42.1	19.4	0.8	46,XY
	31[ζ]	43	47.1	39.9	11.9	1.1	46,XY[β]
	32	44	26.5	51.9	19.8	1.8	46,XY
	33	45	24.3	54.2	18.6	2.9	46,XY
	34	46	31.4	50.6	16.1	1.9	46,XY
		**mean**	**30.5**	**50.5**	**17.4**	**1.6**	
		**median**	**31.4**	**51.4**	**17.3**	**1.4**	
		**min**	**15.6**	**39.9**	**6.6**	**0.8**	
		**max**	**47.1**	**58.1**	**31.7**	**2.9**	
		**n=11**					
**WCB4B**	35[ηζ]	47	36.6	41.6	16.5	5.3	46,XY
	36[ηζ]	48	31.3	57.7	9.7	1.3	46,XY
	37[ηζ]	49	35.2	48.2	13.5	3.1	46,XY
		**mean**	**34.4**	**49.2**	**13.2**	**3.2**	
		**median**	**35.2**	**48.2**	**13.5**	**3.1**	
		**min**	**31.3**	**41.6**	**9.7**	**1.3**	
		**max**	**36.6**	**57.7**	**16.5**	**5.3**	
		**n=3**					
**Total**		**mean**	**30.4**	**53.0**	**14.7**	**1.9**	
		**median**	**31.3**	**53.2**	**14.7**	**1.5**	
		**min**	**15.6**	**39.9**	**6.6**	**0.3**	
		**max**	**47.1**	**65.0**	**31.7**	**5.3**	
		**SD**	**6.3**	**6.4**	**5.2**	**1.1**	
	**n=37**	**n=49**					

[γ]Each expt. represents a single thaw and expansion from designated CyT49 bank
[κ]Transplanted to EFP of SCID/Bg mice, unless indicated
[ζ]Differentiation run that was not transplanted in this analysis
[χ]Independent cytometry analyses
[δ]Performed at d16
[η]Processes selected for the 13C group analysis, n=17 analyses
[λ]20 nuclei examined per analyses. Performed after culture expansion for scaled differentiation
[α]NR: no result, technical failure of analysis
[φ]30 nuclei examined
[β]Analysis performed 7 passages after thaw
