# Supplementary material for: A Scalable System for Production of Functional Pancreatic Progenitors from Human Embryonic Stem Cells
Source: PLoS One. 2012 May 18;7(5):e37004. doi: 10.1371/journal.pone.0037004 (PMC3356395; doi:10.1371/journal.pone.0037004)
Supplement: Table S3 — ELISA samples per in vivo GSIS plot in Figure S8. (PDF) [file pone.0037004.s017.pdf]

Table S3

		wk 5-6, 7 5-8, or 8				wk 8-10, 9-10, or 11				wk 11-15, 15, or 16				wk 16-20, or 16-25				wk 21-50, or 21-43			
		F	5/10	30	60	F	5/10	30	60	F	5/10	30	60	F	5/10	30	60	F	5/10	30	60
A	Total Functioning	10			10	270		12	271	382		282	382	121	24	117	81	159	56	114	73
B	High Function	5			5	197		10	198	272		198	272	106	21	104	69	152	52	110	70
C	Partial Protection	5			5	73		2	73	110		84	110	22	7	17	15				
D	MCB-D	59			60	29		10	198	272		198	272	66	17	64	33	87	26	61	55
E	WCB-D	24			24	36			36	52		30	52	25	4	25	21	65	26	49	15
F	MCB3	16			16	16		16	16	16		16	16								
G	MCB4	23			23	23			23	23		23	23								
H	MCB5	15			15	15			15	15		15	15								
