# Supplementary material for: A Scalable System for Production of Functional Pancreatic Progenitors from Human Embryonic Stem Cells
Source: PLoS One. 2012 May 18;7(5):e37004. doi: 10.1371/journal.pone.0037004 (PMC3356395; doi:10.1371/journal.pone.0037004)
Supplement: Methods S1 — Supplemental methods. (DOC) [file pone.0037004.s018.doc]

## Methods S1: Supplemental Methods

**Culture of hESC in adherent conditions.** XF HA media consisted of DMEM/F12 (Life Technologies, cat#10565) containing GlutaMAX (Life Technologies, cat#10565), supplemented with 10% v/v of Xeno-free KnockOut Serum Replacement (Life Technologies, cat#12618-001), 1% v/v non-essential amino acids (Life Technologies, cat#11140-050), 0.1 mM 2-mercaptoethanol (Life Technologies, cat#21985-023), 1% v/v penicillin/streptomycin (Life Technologies, cat#15070-063), 10 ng/mL heregulin-1β (Peprotech, cat#100-03) and 10 ng/mL activin A (R&D Systems, cat#338-AC). Cells were plated at 50,000 or 33,000 cells/cm^2^ for three and four day growth cycles, respectively. Media volumes were as follows:

| **Vessel** | **60mm** | **T75** | **T175** | **Triple T175** | **2-stack** | **5-stack** |
| --- | --- | --- | --- | --- | --- | --- |
|  | BD Falcon #353004 | BD Falcon #353136 | BD Falcon #353112 | Nunc  #132913 | Corning #3269 | Corning #3319 |
| **SA** | **19.6 cm^2^** | **80 cm^2^** | **175 cm^2^** | **525 cm^2^** | **1272 cm^2^** | **3180 cm^2^** |
| Plating | 4 | 16 | 35 | 105 | 260 | 650 |
| d1 | 5.5 | 22 | 50 | 150 | 350 | 875 |
| d2 | 7 | 28 | 60 | 180 | 450 | 1100 |
| d3 | 8.5 | 35 | 80 | 240 | 550 | 1350 |

Volumes in mL. Pre-feeding prior to passaging (d3 or d4) used the plating volume for each vessel. SA: surface area. Supplier and catalog numbers are indicated.

**Passaging:** On the day of passaging, cultures were fed with fresh growth media and cultured 4-8 hrs before dissociation. Cultures were washed with PBS (Life Technologies, cat#10010-031) and dissociated for 6 mins at 37˚C using pre-warmed Accutase (Innovative Cell Technologies, cat# AT104). In some experiments the Accutase was added then immediately aspirated, such that cell dissociation was achieved in the residual reagent, at a minimal working volume (“Aspirated Accutase”). This approach was employed primarily in cell factories in order to minimize the number of media exchange steps. After a 6-min exposure to Accutase, 3x volume of cold hESC media (without heregulin or activin) was added and the culture was dissociated and collected. Cell suspensions harvested by either of these methods were counted using a ViCell automated cell counter (BD Biosceinces), or a hemocytometer, centrifuged for 5 mins at 200x g and the pellet resuspended in fresh growth media at 1-10x10^6^ cells/mL for subsequent plating. Passaged cells were added to new uncoated vessels, with cell attachment facilitated by including 10% v/v of non-heat inactivated human AB serum (Valley Biomedical, cat#HP1022) for the first day of culture only. A standardized plating volume of 0.2 mL/cm^2^ was used for different tissue culture plates, T-flasks and cell factories. Cultures were fed with fresh media daily, according to the table above.

**Pancreatic differentiation of hESC aggregates in suspension.**

CyT49 aggregates were differentiated to pancreatic progenitors according to the following conditions:

| Time point | Stage | Media Condition | Rotation Speed |
| --- | --- | --- | --- |
| d(-1) | Aggregation | XF HA | 95 |
| d0 | 1 | r0.2FBS-ITS1:5000 A100 W50 | 95 |
| d1 |  | r0.2FBS-ITS1:5000 A100 | 95 |
| d2 | 2 | r0.2FBS-ITS1:1000 K25 IV | 95 |
| d3 |  | r0.2FBS-ITS1:1000 K25 | 95 |
| d4 |  | r0.2FBS-ITS1:1000 K25 | 105 |
| d5 | 3 | db-CTT3 N50 | 105 |
| d6 |  | db-CTT3 N50 | 105 |
| d7 |  | db-CTT3 N50 | 105 |
| d8 | 4 | db-N50 K50 E50 | 105 |
| d9 |  | db-N50 K50 E50 | 95 |
| d10 |  | db-N50 K50 E50 | 95 |
| d11 |  | db-N50 K50 E50 | 95 |
| d12 |  | db-N50 K50 E50 | 95 |
| d13-d16 |  | db-N50 K50 E50 | 95 |

- XF HA: DMEM/F12 (Life Technologies, cat#10565) containing GlutaMAX (Life Technologies, cat#10565), supplemented with 10% v/v of Xeno-free KnockOut Serum Replacement (Life Technologies, cat#12618-001), 1% v/v non-essential amino acids (Life Technologies, cat#11140-050), 0.1 mM 2-mercaptoethanol (Life Technologies, cat#21985-023), 1% v/v penicillin/streptomycin (Life Technologies, cat#15070-063), 10 ng/mL heregulin-1β (Peprotech, cat#100-03) and 10 ng/mL activin A (R&D Systems, cat#338-AC).
- r0.2FBS: RPMI 1640 (Mediatech, cat#15-040-CV), 0.2% FBS (HyClone, cat#SH30070.03), 1x GlutaMAX-1 (Life Technologies, cat#35050-061), 1% v/v penicillin/streptomycin.
- ITS: Insulin-Transferrin-Selenium (Life Technologies, cat#41400-045) diluted 1:5000 or 1:1000.
- A100: 100 ng/mL recombinant human Activin A (R&D Systems, cat#338-AC).
- W50: 50 ng/mL recombinant mouse Wnt3A (R&D Systems, cat#1324-WN).
- K25: 25 ng/mL recombinant human KGF (R&D Systems, cat#251-KG).
- IV: 2.5 μM TGF-β RI Kinase inhibitor IV (EMD Bioscience, cat#616454).
- db: DMEM HI Glucose (HyClone cat#SH30081.01) supplemented with 0.5x B-27 Supplement (Life Technologies, cat#17504-044), 1x GlutaMAX-1 and 1% v/v penicillin/streptomycin.
- CTT3: 0.25 μM KAAD-Cyclopamine (Toronto Research Chemicals, cat#K171000) and 3 nM TTNPB (Sigma-Aldrich, cat#T3757).
- N50: 50 ng/mL recombinant human Noggin (R&D Systems, cat#3344-NG).
- K50: 50 ng/mL recombinant human KGF (R&D Systems, cat#251-EG).
- E50: 50 ng/mL recombinant human EGF (R&D Systems, cat#236-EG).

**Suspension culture of undifferentiated hESC aggregates.**

Suspension aggregates of hESC could be formed and cultured in one of the following hESC medium: XF HA, StemPro, SP HAI (formulations described in the methods, and hESC methods above), the defined media described previously [Wang *et al*, Blood 110: 4111-4119 (2007)], or mTeSR1 (Stem Cell Technologies, cat# 05850). Aggregates were formed essentially as described for the differentiation process. On the day of passaging, adherent cultures were fed with fresh growth media and cultured 4-8 hrs before dissociation. Cultures were washed with calcium/magnesium-free PBS (Life Technologies, cat#10010-031) and dissociated for 6 mins at 37˚C using pre-warmed Accutase (Innovative Cell Technologies, cat# AT104). Dissociated cells were gently collected using 3x volume of cold hESC media (without heregulin or activin), counted using a hemocytometer, centrifuged for 5 mins at 200x g and the pellet resuspended in fresh hESC media at 1-10x10^6^ cells/mL. Cells were then seeded into ultra-low adhesion 6-well trays (Greiner BioOne, cat#657185) at 1x10^6^ cells/mL in hESC media and 5.5 mL per well. The 6-well trays were incubated at 37˚C on orbital rotators set at 95 rpm (Innova2000, New Brunswick Scientific). During overnight culture, single cells aggregated to form spherical clusters approximately 100-200 μm in diameter and roughly 5,000 aggregates per well. For expansion of undifferentiated cell cultures in suspension, each well was fed with fresh hESC media daily for 3-5 days as required. For serial passaging experiments, hESC aggregates were cultured in XF HA, StemPro, SP HAI, or the defined media described previously [Wang *et al*, Blood 110: 4111-4119 (2007)]. For passaging, undifferentiated hESC aggregates along with culture medium from one well of a 6-well culture dish were transferred to a 15 mL tube. Multiple wells were pooled when necessary to simplify passaging. The aggregates were allowed to settle by gravity and most of the medium was aspirated. The aggregates were washed once with calcium/magnesium-free PBS, settled by gravity and most of the PBS was aspirated. An equal volume, or greater, of pre-warmed Accumax dissociation reagent (Sigma, cat#A7089) was added to the aggregate pellet and incubated for approximately 10 mins, triturating periodically with a 5 mL pipette to promote dissociation. Once the aggregates were reduced to small clumps, a 3x volume of wash medium (DMEM/F12, containing 0.2% Cohn fraction V BSA (Millipore, cat#82-047-3)) was added, and trituration was continued until a single-cell suspension was generated. The cells were then counted using a hemocytometer, centrifuged for 5 mins at 200x g and the pellet resuspended in fresh hESC media at 1-10x10^6^ cells/mL. Cells were then seeded into ultra-low adhesion 6-well trays (Greiner BioOne, cat#657185) for aggregation and culture as described above.

**Antibodies used for cell culture immunofluorescence, cytometry and immunocytochemistry of aggregates and grafts.**

| **Primary Antibodies** | **Species** | **Dilution** | | **Supplier** | **cat#** |
| --- | --- | --- | --- | --- | --- |
| *[2˚ antibodies for cytometry]* |  | **Flow** | **ICC** |  |  |
| Anti-PDX1 | Guinea Pig |  | 1:500 | Abcam | ab 47308 |
| Anti-PDX1  *[2˚: Donkey Anti-goat-DL649]*^∫^ | Goat | 1:2000  *[1:6000]* |  | Abcam  *[Jackson IRL^¶^]* | ab 47383 |
| Anti-NKX6-1  *[2˚: Donkey Anti-mouse-PE]*^∫^ | Mouse | 1:750  *[1:500]* | 1:100 | DSHB*  *[Jackson IRL^¶^]* | F55A12 |
| Anti-chromogranin  *[2˚: Donkey Anti-Rabbit-AF488]*^∫^ | Rabbit | 1:1000  *[1:500]* | 1:200 | DAKO  *[Life Tech^§^]* | A0430 |
| Anti-SOX17 [AF488] | Mouse | 1:10 |  | BD Biosciences | 562205 |
| Anti-FOXA2 (HNF3β [R-PE] | Mouse | 1:10 |  | BD Biosciences | 561589 |
| Anti-insulin | Guinea Pig |  | 1:500 | DAKO | A0564 |
| Anti-somatostatin | Rabbit |  | 1:500 | DAKO | A0566 |
| Anti-glucagon | Mouse |  | 1:500 | Sigma | G2654 |
| Anti-FOXA2 (HNF3β | Rabbit |  | 1:500 | Cell Signaling | 31-43S |
| Anti-FOXA2 (HNF3β | Goat | 1:40 |  | SC Biotech** | SC-6554 |
| Anti-NKX2-2 | Mouse |  | 1:50 | DSHB* | 74.5A5 |
| Anti-CDX2 | Mouse | 1:50 | 1:50 | Abcam | ab15258 |
| Anti-CDX2 [AF647]^∫^ | Mouse | 1:100 |  | BD Biosciences | 560395 |
| Anti-cytokeratin19 | Rabbit |  | 1:500 | Abcam | ab52907 |
| Anti-trypsin | Rabbit |  | 1:200 | Biodesign | K50900R |
| Anti-OCT4 | Mouse |  | 1:100 | SC Biotech** | SC-5279 |
| Anti-OCT4 [R-PE]^∫^ | Mouse | 1:10 |  | BD Biosciences | 560186 |
| Anti-Nanog | Goat |  | 1:100 | R&D Systems | AF1997 |
| Anti-TRA-1-60 | Mouse |  | 1:100 | Millipore | MAB4360 |
| Anti-TRA-1-81 | Mouse |  | 1:100 | Millipore | MAB4381 |
| Anti-SSEA4 | Mouse |  | 1:100 | Millipore | MAB4304 |
| Anti-SSEA4 [AF488]^∫^ | Mouse | 1:10 |  | eBiosciences | 53-8843 |
| Anti-human nuclei [AF568]^∫^ | Mouse |  | 1:250 | Millipore | MAB1281 |
| Secondary Antibodies |  |  |  |  |  |
| Anti-guinea pig [Cy5]^∫^ | Donkey |  | 1:500 | Jackson IRL^¶^ | 706-176-148 |
| Anti-goat, Anti-mouse, Anti-rabbit, Anti-guinea pig [AF488, or 555]^∫^ | Donkey or Goat | 1:500 to 1:6000 | 1:500 to 1:1000 | Life Tech^§^ | Various |
| Anti-rabbit [DL488]^∫^ | Donkey | 1:1000 |  | Jackson IRL^¶^ | 711-486-152 |
| Anti-mouse [R-PE]^∫^ | Donkey | 1:500 |  | Jackson IRL^¶^ | 715-116-151 |
| Anti-goat [Cy5]^∫^ | Donkey | 1:6000 |  | Jackson IRL^¶^ | 705-176-147 |

*DSHB: Developmental Studies Hybridoma Bank. **Santa Cruz Biotech. ^¶^Jackson Immuno Research Laboratories. ^§^Life Technologies (Molecular Probes). Secondary antibodies were obtained from Jackson Immunoresearch, Life Technologies, and BD Biosciences. ^∫^Conjugated with: AlexaFluor (AF) 488, 555, 647, or 594; Cyanine5 (Cy5), R-phycoerythrin (R-PE), DyLight 488 (DL488), DyLight 649 (DL649).

**Quantitative RT-PCR (qPCR) Analysis**

Total RNA was isolated from samples of typically 5x10^6^ cells using the RNeasy Mini kit (Qiagen, cat# 74104) and cDNA synthesis was performed from approximately 1 µg RNA using the iScript cDNA Synthesis kit (Bio-Rad, cat# 170-8891) according to the manufacturer’s instructions. Real time qPCR reactions were prepared using the SYBR Green PCR master mix (Applied Biosystems, cat# 4309155) and were run using the Opticon 2 real time PCR detection system (Bio-Rad, cat# CFB-322001G). Triplicate qPCR data were normalized against three housekeeping genes (GUSB, CYCG and TBP) using geometric averaging [Vandesompele *et al*, Genome Biology 18;3(7):RESEARCH0034 (2002)] and plotted relative to the left-most sample. Primers were as follows (all sequences 5'-3'):

Qiagen QuantiTect primer assays: Nanog, SOX2, Eomes, SOX17.

POU5F1 (F): TGGGCTCGAGAAGGATGTG (R): GCATAGTCGCTGCTTGATCG

MIXL1 (F): CCGAGTCCAGGATCCAGGTA (R): CTCTGACGCCGAGACTTGG

CXCR4 (F): CACCGCATCTGGAGAACCA (R): GCCCATTTCCTCGGTGTAGTT

HNF1B (F): TCACAGATACCAGCAGCATCAGT (R): GGGCATCACCAGGCTTGTA

HNF4A (F): CATGGCCAAGATTGACAACCT (R): TTCCCATATGTTCCTGCATCAG

GUSB (F): ACGCAGAAAATATGTGGTTGGA, (R): GCACTCTCGTCGGTGACTGTT

CTCG (F): CTTGTCAATGGCCAACAGAGG, (R): GCCCATCTAAATGAGGAGTTGGT

TBP (F): TGTGCACAGGAGCCAAGAGT, (R): ATTTTCTTGCTGCCAGTCTGG
